# Metabolomic analysis of tomato seed germination

**DOI:** 10.1007/s11306-017-1284-x

**Published:** 2017-10-23

**Authors:** Rashid H. Kazmi, Leo A. J. Willems, Ronny V. L. Joosen, Noorullah Khan, Wilco Ligterink, Henk W. M. Hilhorst

**Affiliations:** Wageningen Seed Lab, Lab. of Plant Physiology, Wageningen University, Droevendaalsesteeg 1, 6708 PB Wageningen, The Netherlands

**Keywords:** Canonical correlation analysis (CCA), GC-TOF/MS, Generalized genetical genomics (GGG), Metabolomics, mQTL analysis, Seed performance, *Solanum lycopersicum*, *Solanum pimpinellifolium*, Tomato

## Abstract

**Introduction:**

Seed germination is inherently related to seed metabolism, which changes throughout its maturation, desiccation and germination processes. The metabolite content of a seed and its ability to germinate are determined by underlying genetic architecture and environmental effects during development.

**Objective:**

This study aimed to assess an integrative approach to explore genetics modulating seed metabolism in different developmental stages and the link between seed metabolic- and germination traits.

**Methods:**

We have utilized gas chromatography-time-of-flight/mass spectrometry (GC-TOF/MS) metabolite profiling to characterize tomato seeds during dry and imbibed stages. We describe, for the first time in tomato, the use of a so-called generalized genetical genomics (GGG) model to study the interaction between genetics, environment and seed metabolism using 100 tomato recombinant inbred lines (RILs) derived from a cross between *Solanum lycopersicum* and *Solanum pimpinellifolium*.

**Results:**

QTLs were found for over two-thirds of the metabolites within several QTL hotspots. The transition from dry to 6 h imbibed seeds was associated with programmed metabolic switches. Significant correlations varied among individual metabolites and the obtained clusters were significantly enriched for metabolites involved in specific biochemical pathways.

**Conclusions:**

Extensive genetic variation in metabolite abundance was uncovered. Numerous identified genetic regions that coordinate groups of metabolites were detected and these will contain plausible candidate genes. The combined analysis of germination phenotypes and metabolite profiles provides a strong indication for the hypothesis that metabolic composition is related to germination phenotypes and thus to seed performance.

**Electronic supplementary material:**

The online version of this article (doi:10.1007/s11306-017-1284-x) contains supplementary material, which is available to authorized users.

## Introduction

Genomic approaches have accelerated the study of the quantitative genetics that underlie phenotypic variation. The mutualistic relationship between metabolomics and genetics goes back to Mendel’s reliance upon metabolic phenotypes (anthocyanins and starch) to develop his basic genetic theory (Kliebenstein 2009). The understanding of DNA structure and metabolism was further enhanced as genetics has played an equally important role in the reconstruction of biochemical pathways eventually shaping our current understanding of gene regulation (Ruggieri et al. 2016). The combination of metabolomics and genetics has provided powerful insights into the origin and maintenance of natural variation (Keurentjes et al. 2006). Given their huge diversity, metabolites can be associated with specific genetic markers, mRNA transcripts, and enzyme activities, allowing a linkage between variation from genetic to biochemical levels that is more complex for less-defined or more pleiotropic phenotypes, such as seed performance (Joosen et al. 2013a; Keurentjes and Sulpice 2009; Keurentjes et al. 2008; Koornneef et al. 2004; Rosental et al. 2016).

Variations in plant growth, as well as in seed and metabolic traits, have been detected for a series of natural accessions and recombinant inbred lines (Joosen et al. 2013a, b; Meyer et al. 2007; Prinzenberg et al. 2010; Rosental et al. 2016; Schauer et al. 2006; Skogerson et al. 2010; Toubiana et al. 2012, 2015). Although only weak relationships have been suggested between growth and the levels of individual metabolites (Meyer et al. 2007), highly significant links between biomass and specific combinations of metabolites have been demonstrated (Lisec et al. 2008; Prinzenberg et al. 2010). Metabolite profiling in Arabidopsis during seed development (Fait et al. 2006; Toubiana et al. 2012) identified major metabolic abundance switches associated with successive developmental stages. Although certain alterations that impair cellular structures and metabolism have been implicated in seed deterioration, the molecular and biochemical basis of seed performance is not well understood.

The combination of metabolomics with quantitative genetics is at the heart of our understanding of biochemical phenotypes (Reed et al. 2017). Correspondingly, the fitness consequences of these metabolic changes are an important component in the determination of the genetic architecture of species, making metabolomics unique in the quest for system-wide coverage of all metabolites (Kliebenstein 2009; Phillips 2008). Research has consistently shown that quantitative metabolomics data can directly be mapped onto the metabolic network, ultimately opening the door for identification of metabolic reactions, networks and biochemical pathways (Joosen et al. 2013a; Keurentjes 2009; Rowe et al. 2008; Sulpice et al. 2010; Toubiana et al. 2016). Several studies have demonstrated the use of metabolic QTLs (mQTLs) in integration of different levels of genomic information (sequence, transcript, and protein) to understand plant and seed phenotypes better, improve crop breeding and obtain ecological inference about the corresponding selective pressure acting on these QTLs (Alseekh et al. 2015; Basnet et al. 2016; Lisec et al. 2008, 2009; Matsuda et al. 2012; Reed et al. 2017; Schauer et al. 2008, 2006; Toubiana et al. 2015).

Genetical genomics approach brings together traditional QTL mapping with gene expression, protein and metabolic profiling studies for a better understanding of the genetic mechanisms influencing complex traits (Jansen and Nap 2001; Joosen et al. 2013b). This is a useful methodology in studying molecular perturbation in biological systems and several studies have used this approach, focusing on natural variation (Keurentjes et al. 2006), the connection between metabolism and yield-associated traits or biomass (Meyer et al. 2007; Schauer et al. 2006), and the identification of metabolic quantitative trait loci (mQTL) (Alseekh et al. 2015; Lisec et al. 2008; Rosental et al. 2016; Toubiana et al. 2015). Most studies using genetical genomics have been carried out in *Arabidopsis thaliana* mainly due to the availability of high quality mapping populations and the commercially available genome-wide micro-arrays where several studies in various RIL populations have indicated extensive genetic regulation of gene expression (Cubillos et al. 2012; Keurentjes et al. 2007; Lowry et al. 2013; Snoek et al. 2012; West et al. 2007). However, little attention has been paid to tomato, in particular with respect to seed performance evaluation. In addition to molecular networks, the genetic perturbations of biological systems also depend upon environmental conditions and, thus, a comprehensive understanding of biological systems requires studying them across multiple environments.

We applied a generalized genetical genomics (GGG) approach for metabolic profiling using GC-TOF/MS on 100 recombinant inbred lines (RILs) of tomato to describe the genetic regulation of variation in the tomato seed metabolome. This new GGG model may prove to be useful in tomato seeds and allows the investigation of the mechanisms that contribute to complex variations in the tomato seed metabolome during germination by analyzing two different developmental stages in one study and it offers unique reduction of experimental load with minimal compromise in statistical power as has been shown for Arabidopsis seed germination and associated metabolites (Joosen et al. 2013a). Germination efficiency is affected by reserve accumulation during seed development or their mobilization during seed germination as well as several unknown factors (Fait et al. 2006; Rosental et al. 2014). To elucidate the nature of such factors, we analyzed the metabolite content of tomato seeds at two developmental stages: dry mature and 6 h-imbibed seeds. Metabolic fluxes are arrested in the dry seed; however, upon imbibition the dry seed rapidly resumes metabolic activity (Bewley et al. 2013; Rosental et al. 2014). We chose the 6 h stage for optimum synchronization of seed germination as full rehydration of dry seeds typically completes in less than 2 h, and assuming that many metabolic processes will have started after 6 h of imbibition. Thus, it was essential to make an intelligent selection of the time point for imbibed seeds as germination extends from the onset of imbibition in an environment meeting the normal physiological requirements for germination to the inception of cell division and elongation (Bewley et al. 2013).

The application of a GGG model, which is a systems genetics approach, provides a broad overview of changes in primary metabolic processes that occur during dry and imbibed tomato seed developmental stages. In particular, it takes into account genetics and chosen environmental perturbations (different seed developmental stages, i.e. dry and imbibed seeds) in combination with the analysis of the genetic variation present in the studied RILs, to study the multiple environments and to identify genotype-by-environment interactions, offering a unique reduction of experimental load with minimal compromise of statistical power. Thus, the present approach reveals the plasticity of molecular networks in tomato for seed performance traits and forms a crucial step toward understanding different influences of genetic and developmental responses in tomato seeds. The present study attempts to link seed traits to metabolic signatures. Furthermore, it supports previous findings in other crops and provides additional evidence that relationships between a seed trait and a single metabolite is generally absent but that strong canonical correlation with a specific combination of metabolites illustrates the complexity of such quantitative traits (Meyer et al. 2007).

## Materials and methods

### Growth conditions and seed collection

A *Solanum lycopersicum* × *S. pimpinellifolium* F_8_ RIL population of 100 lines was used that has been genotyped with 5529 SNP markers of which 865 unique markers were used for mapping (Kazmi et al. 2011).The population was grown twice under controlled conditions in the greenhouse facilities at Wageningen University, in the Netherlands from January to June 2009. Seeds were sown on soil and after 2 weeks two plants from each RIL were planted in a 100 × 100 mm Rockwool block (MM100/100, Grodan B.V.). The day and night temperatures were maintained at 25 and 15 °C, respectively, with 16 h light and 8 h dark (long-day conditions). All the RILs were uniformly supplied with the basic dose of fertilizer (Supplemental Table 1). Seeds were extracted from healthy fruits and treated with 1% hydrochloric acid (HCl) to remove the pulp sticking onto the seeds. The solution of tomato seed extract with diluted HCl was passed through a fine mesh sieve and washed with water to remove the remaining parts of the pulp and remnants of HCl. The seeds were processed and disinfected by soaking in a solution of trisodium phosphate (Na_3_PO_4_·12H_2_O) for 1 h. Finally, the seeds were dried on clean filter paper at room temperature and were brushed to remove impurities with a seed brusher (Seed Processing Holland BV, Enkhuizen, The Netherlands, http://www.seedprocessing.nl). The cleaned seeds were dried for 3 days at 20 °C and were stored in a cool, dry storage room (13 °C and 30% RH) in paper bags until further use.

### Generalized genetical genomics (GGG)

In this study the population of 100 RILs was intelligently allocated to two sub-populations optimized for the distribution of parental alleles in such a way that the allele distribution in the two sub-populations is as similar to each other and to the total population as possible using the R-procedure DesignGG (Joosen et al. 2013a; Li et al. 2009); hence 50 RIL lines were used for dry seeds and 50 lines for 6 h imbibed seeds (Supplemental Fig. 1). DesignGG is applicable to linkage analysis of experimental crosses, e.g. recombinant inbred lines, as well as to association analysis of natural populations (Joosen et al. 2013a; Li et al. 2008, 2009). DesignGG allows users to intelligently select and allocate individuals to experimental units and conditions such as drug treatment (Li et al. 2009). The user can maximize the power and resolution of detecting genetic, environmental and interaction effects in a genome-wide or local mode by giving more weight to genome regions of special interest.

### Extraction, derivatization, and analysis of seed metabolites using GC-TOF/MS

In December 2009, a bulk of approximately 70–100 seeds (30 mg) were either immediately frozen in liquid nitrogen or imbibed for 6 h in the dark on pre-wetted filter paper (ft-30303-85, Sartorius) with demineralized water, after which seeds were frozen in liquid nitrogen. The extraction method is modified from the method previously described by Roessner et al. (2000). A bulk of approximately 70–100 seeds (30 mg) was homogenized in 2 ml tubes (Eppendorf) with two iron balls (2.5 mm), pre-cooled in liquid nitrogen. For the homogenization the micro-dismembrator (Sartorius) was used at 1500 rpm. A solution of 700 µl methanol/chloroform (4:3, Biosolve) with a standard (0.2 mg/ml ribitol) was added and mixed thoroughly. After 10 min sonication, 200 µl double distilled and filtered water (MilliQ, Millipore) was added to the mixture followed by vortexing and centrifuging (5 min 13,500 rpm). The methanol phase was collected in a glass vial (98213, Grace) and 500 µl methanol/chloroform was added to the remaining organic phase and kept on ice for 10 min. 200 µl MilliQ was added followed by vortexing and centrifuging (5 min 13,500 rpm). Again, the methanol phase was collected and mixed with the other collected phase. 100 µl, applied to a vial with an insert (06090357, Grace) was dried overnight in a speedvac (35 °C Savant SPD121). The GC-TOF/MS method was previously described by Carreno-Quintero et al. (2012). Briefly, dried samples were crimp capped with a magnetic cap (8618261, Grace) in the presence of argon to prevent reaction with H_2_O. Samples were derivatized online using a Combi PAL auto sampler (CTC Analytics). 12.5 µL of *O*-methylhydroxylamine hydrochloride (20 mg ml^− 1^ pyridine) was added to the dried samples an incubated for 30 min. Then the samples were derivatized with 17.5 µL *N*-methyl-*N*-trimethylsilyltrifluoroaceticamide (69479, Sigma) for 60 min. An alkane mixture of C10–C34 was added to calculate the retention indices of metabolites. 2 µL of the derivatized samples were injected into an Optic three high-performance injector (ATAS) at 70 °C at a spilt ratio of 19:1 and the injector was heated to 240 °C at 6 °C/s^− 1^. Chromatography was performed on a Agilent 6890 gas chromatograph (Agilent Technologies) coupled to a Pegasus III time-of-flight mass spectrometer (Leco Instruments) using a VF-5 ms capillary column (30 m × 0.25 mm × 0.25 µM, Varian) including a 10 m guardian column with helium as carrier gas with a flow rate of 1 ml/min^− 1^. The oven was isothermal for 2 min at 70 °C followed by a 10 °C/min^− 1^ ramp to 310 °C. The transfer line was set at 270 °C and the column effluent was ionized by electron impact at 70 eV. Solvent delay was set at 300 s. Detector voltage at 1600 V.

### Data processing

Raw data was processed using the ChromaTOF software 2.0 to obtain netCDF files. Signal to noise ratio was set to 5. Further processing was done by Metalign software (Lommen 2009), to extract and align the mass signals. Baseline correction was done with a, peak slope factor (x Noise) set to 1, a peak threshold factor (x Noise) of 2 and a peak threshold of 25 with an average peak width at half height of 25 (scans). Peaks were aligned with a maximum shift of 50 scans. This resulted in 60,745 different mass signals. This output was loaded in Metalign Output Transformer (METOT; Plant Research International, Wageningen) and the mass signals that were present in less than three RILs or lower than 35 were discarded. Remaining peaks below background were randomized from 50 to 100%. Out of all the remaining mass signals (5601), centrotypes were formed using the MSclust program (Tikunov et al. 2011) with the following parameters: correlation threshold at 0.9 with 0.01 margin softness; PDF correlation of 0.8 with margin of 0.02; a peak width of 20 with a margin of 4 and Criterion was stopped at two masses. This resulted in 167 unique centrotypes (representative masses). The mass spectra of these centrotypes were used for identification by matching to an in-house constructed library and the NIST05 [National Institute of Standards and Technology, Gaithersburg, MD, USA; http://www.nist.gov/srd/mslist.htm) and Golm libraries (http://csbdb.mpimp-golm.mpg.de/csbdb/gmd/gmd.html)]. This identification is based on similarity of spectra and comparison with retention indices calculated by using a third order polynomial function (Strehmel et al. 2008). Details can be found in Supplemental Tables 2 and 3.

### Statistical analysis of GC-TOF/MS data

Metabolomics data were log2 transformed and then statistically analyzed using the rank product method (Breitling et al. 2004) to identify differentially changed metabolites with the Bioconductor ‘RankProd’ package. Significantly changed metabolites showed a false discovery rate (FDR) < 0.05. The FDR value in the rank product was obtained with 1000 random permutations. Principal component analysis was performed on the data sets obtained from metabolite profiling with the R “prcomp” package. The data were log transformed and normalized to the median of the entire sample set for each metabolite before analysis. This transformation reduces the influence of outliers. Heat map presentation and clustering were performed with Spearman correlation coefficient matrices. R-packages “MASS”, “Hmisc”, “VGAM” and their presentation as heat maps using R-packages “gplots” and “graphics” were used. Also ANOVA was performed using R statistics (http://www.r-project.org/) with 5% FDR correction.

### QTL analysis

For QTL analysis a previously developed R script (Joosen et al. 2013a) was used, which uses functions and data structures from the R/qtl package (Arends et al. 2010; Broman et al. 2003) to enable mapping of the observed trait variation while taking the different developmental stages into consideration. The developed R script uses a linear model to calculate the likelihood of genotype-to-phenotype linkage for each marker with the following model:

$${{\text{y}}_{\text{i}}}={\beta _0}+{\beta _1}{{\text{e}}_{\text{i}}}+{\beta _2}{{\text{g}}_{\text{i}}}+{\beta _3}{{\text{e}}_{\text{i}}}:{{\text{g}}_{\text{i}}}+{\varepsilon _{\text{i}}}$$

where y_i_ is the i^th^ observation of the studied phenotype, variable g_i_ is the genotype, e_i_ is a vector with seed conditions, and g_i_:e_i_ the interaction term. The values β_j_ represent parameters to be estimated, and ε_i_ is the error term. The simplified description (Y = E + G + G:E + ε) of this linear model will be used henceforward. Separate likelihood estimates [− log probability, henceforth log of the odds (LOD) scores] are generated for the E, G, and G:E effects.

Data was pre-processed using a log2 transformation and per phenotype outliers were removed after Z-transformation (Z-scores > 3). With the open source statistical package R (version 2.14.1) we fitted a basic linear model (y_i_ = β_0_ + β_1_g_i_ + ε_i_) on the two conditions separately. This was followed by a combined mapping allowing for a developmental co-variate and interaction term between the genetic marker and the developmental stage (y_i_ = β_0_ + β_1_e_i_ + β_2_g_i_ + β_3_e_i_:g_i_ + ε_i_) (Joosen et al. 2013a). P-values from all mappings were transformed into LOD scores by taking the –log10. Additionally, raw and normalized effects were calculated for each individual environment. Normalized effects were calculated by dividing the difference between the maximum and minimum values for that trait by the mean effect at the marker. LOD significance was determined using permutations for the combined mapping of the two environments: a LOD score of 3.0 was found to be significant (Breitling et al. 2008).

### Integrated analysis of phenotypic and metabolite data

The relationship between seed performance phenotypes and metabolite profiles was measured by simple Spearman correlation between the seed performance phenotypes and relative abundances of all metabolites, and by a more complex multiplicative model (Meyer et al. 2007). Missing values in the metabolite matrix were imputed with a self-organizing map (SOM) algorithm using R package “SeqKnn”.

### Canonical correlation analysis (CCA)

Canonical correlation analysis calculates the highest possible correlation between linear combinations of the columns from two matrices with the same number of rows. The R function “cancor” was used to calculate the canonical correlation between metabolites and seed performance phenotypes. For cross validation a partial least square (PLS) regression was performed. To carry out the procedure the R package “pls” implementing partial least squares regression (PLSR) was used (http://www.r-project.org). All procedures were applied after the missing value estimation followed by normalization of the metabolic matrix.

### Network analysis and graph clustering

A matrix of correlation between all trait pairs was generated. Initially the R-package “igraph” was used to visualize the network and then we exported the graph to a file which can be read by DPClus (Altaf-Ul-Amin et al. 2006; Csardi and Nepusz 2006; Fukushima and Nishida 2016). Essentially, this algorithm divides the network into modules or groups of vertices that are more connected between themselves than to nodes from others and extract densely connected nodes as a cluster. In this study, we used the overlapping-mode with the DPClus settings since the overlapping-mode is consistent with the overlap of many of the metabolic pathways and protein complexes. The algorithm of DPClus receives three inputs: the network, a value of minimum density we allow for the generated clusters (d_in_) and a minimum value for cluster property that determines the nature of periphery tracking (cp_in_). The values for density and cluster properties should be within the following range: 0 < d_in_ ≤1, and 0 < cp_in_ ≤1 (Altaf-Ul-Amin et al. 2006). We set the parameter settings of cluster property cp; density values were set to 0.5 as it gives the best performance in a graph clustering.

## Results and discussion

Approaches employing transcriptomics, proteomics, and metabolomics have yielded vast data sets, allowing the correlation of physiological states with patterns of gene expression, protein levels, and metabolite abundance. Omics studies in general are often expensive and laborious, particularly to incorporate developmental and environmental perturbation. To address this challenge, generalized genetical genomics (GGG) as an alternative experimental setup using balanced fractions of a RIL population has been used recently for genetic and environmental perturbation (Joosen et al. 2013a; Li et al. 2008). This enables a cost-effective experimental setup for hypothesis-generating research in multiple environments (Joosen et al. 2013a; Li et al. 2008). Furthermore, analysis and interpretation of omics data at multiple layers and delivering models of causation is also cumbersome. Progress made in analytical and statistical techniques now enables the construction of regulatory networks that integrate the various levels of biological information, including transcriptional and (post) translational regulation, as well as metabolic signalling pathways (Serin et al. 2016).

### Metabolite distribution and detection

We utilized an in-house gas chromatography–time of flight–mass spectrometry (GC-TOF/MS) metabolomics platform to measure metabolite accumulation in the seeds of a *S. lycopersicum* (Moneymaker ‘MM’) × *Solanum pimpinellifolium* (‘Pimp’) RIL population (Voorrips et al. 2000). This GC-TOF/MS platform detects predominantly primary metabolites, and metabolites are identified based on comparison with reference spectra [an in-house constructed library and the NIST (National Institute of Standards and Technology, Gaithersburg, MD, USA; http://www.nist.gov/srd/mslist.htm) and Golm libraries (http://csbdb.mpimp-golm.mpg.de/csbdb/gmd/gmd.html)]. In total, 167 metabolites were detected in this study and the chemical nature was identified for 66 of these metabolites. The known metabolites included central metabolism derived compounds, such as glucose-6-phosphate, members of the tricarboxylic acid (TCA) cycle, such as succinate, citrate and malate, members of the membrane/phospholipid biosynthesis, such as glycerol-3-phosphate, ethanolamine, amino acids and precursors thereof, sugars, and some other common metabolic end products (Supplemental Table 2). This list was compiled to encompass the different classes of intermediates in primary metabolism. These metabolites are ubiquitously present in living organisms and are at the core of the biochemical reaction networks with the largest fluxes and largest number of regulatory circuits.

The majority of the metabolites were detected in both parents and in more than 90% of the RILs. Transgressive segregation for metabolite presence was manifested in a significant fraction of the metabolites found in the RIL population. Analysis of the RILs for 167 metabolites identified positive and negative transgressive segregation for metabolite accumulation (Supplemental Fig. S2). Thus, *S. lycopersicum* and *S. pimpinellifolium* possess significant genetic variation for metabolite accumulation.

**Fig. 1:**
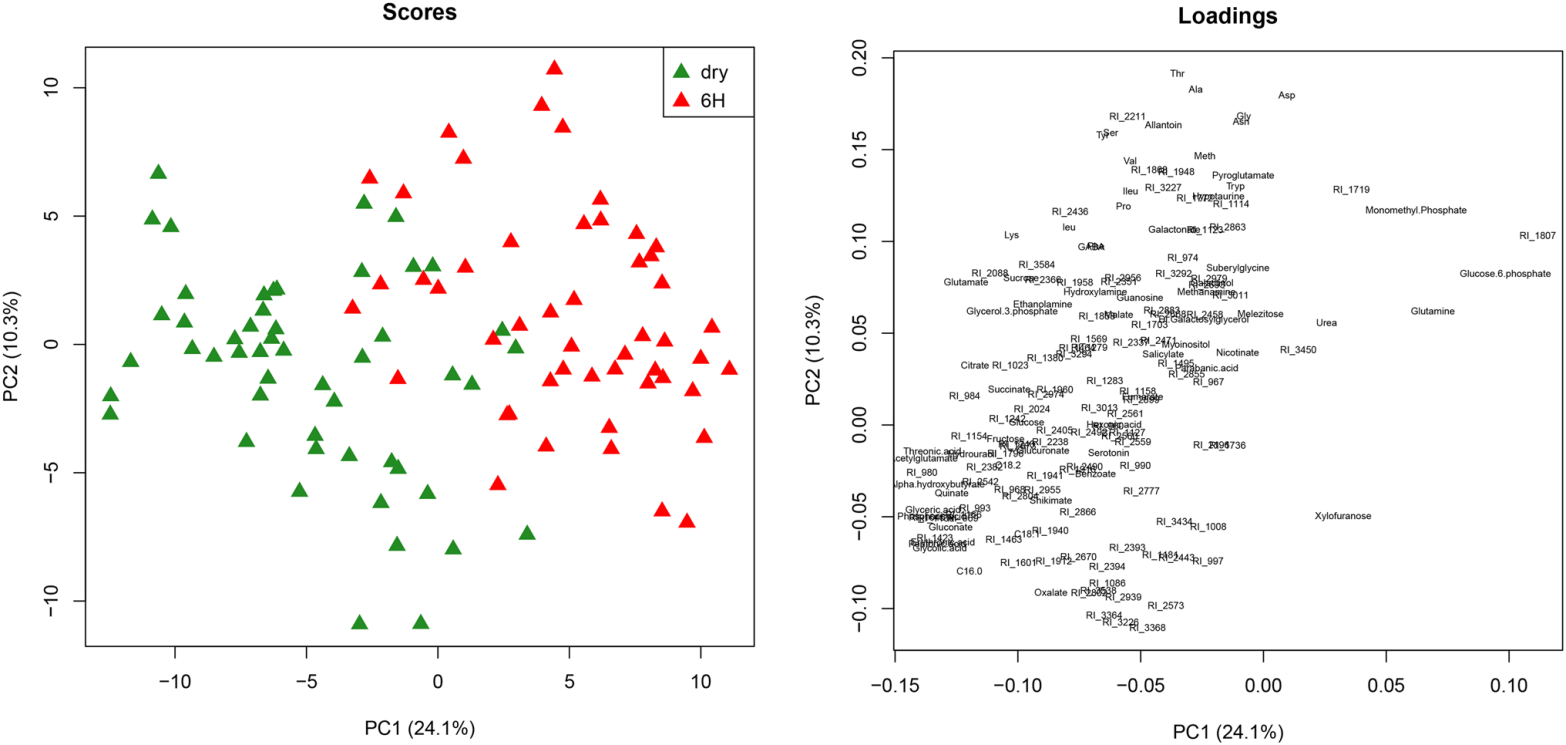
Principal component analyses of all detected metabolites for dry and 6 h imbibed tomato seeds. Symbols: green rectangles, dry seeds; red rectangles, imbibed. Each data point corresponds to the analysis of one of 100 genotypes. Scores of principal component analysis are presented for dry and 6 h based on a combination of 2 components (PC1 and 2) (left). Variances of 24.1% for PC1 and 10.3% for PC2 were recorded in each component. Loading scores of metabolites are presented for PC1 and PC2 (*right*)

The data set obtained by GC-TOF/MS for the RIL population was examined by principal component analysis (Fig. 1). Principal component analysis of the metabolic profiles revealed the internal structure in the data, showing that the first component clearly separates dry seeds and 6-hour imbibed seeds, explaining 24.1% of the total variation (Fig. 1). To confirm the most important principal components (PCs) of these samples we prepared score plots for the dataset. The loading plots highlighted and visualized metabolites with a significant role in seed developmental stage separation (Fig. 1).

### Coordinated changes of metabolites in dry and 6 h tomato seeds

**Fig. 2:**
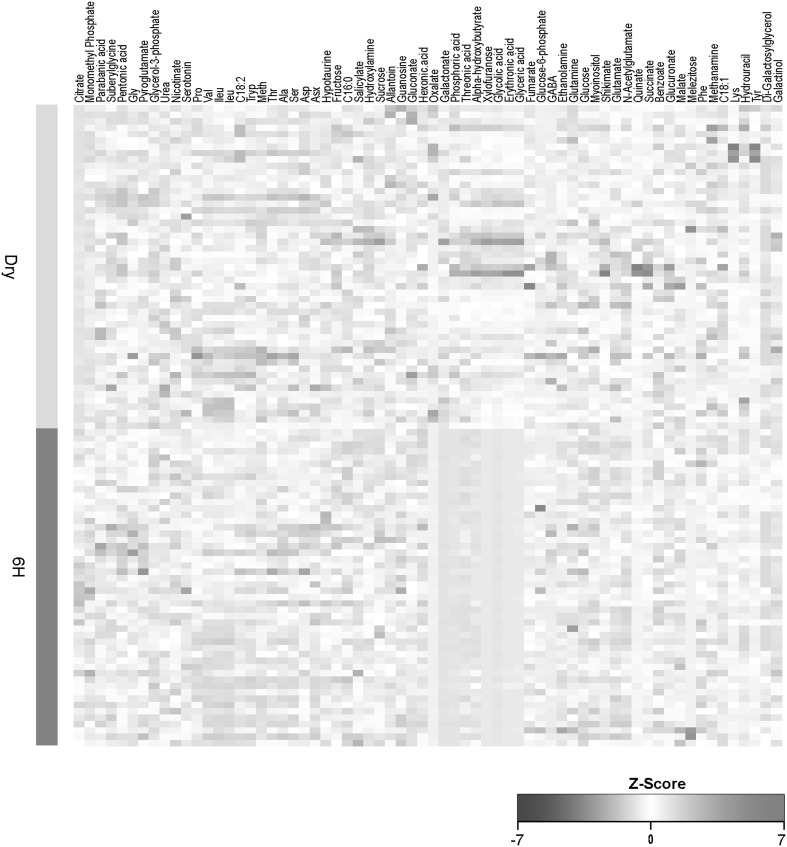
Metabolite profiles in dry and 6 h imbibed seeds of tomato. Metabolite levels between dry and 6 h seed developmental stages were compared. The vertical Green and Red bar colors represent variability of metabolite abundance between F_8_ recombinant inbred lines (RILs) for dry and 6 h. The relative abundance of each metabolite represent the mean of all genotypes (n = 100). A hierarchical classification of metabolites was done according to a dissimilarity scale using the distance function 1-correlation. The dissimilarity index is employed for cluster analysis to arrange different metabolites according to their similarity. Z-values of measurements are color-coded as indicated in the scale on the bottom of the heat map, from blue to red

Quantitative changes in the amounts of major metabolites in the two different stages are presented in Fig. 2. The progression of seeds from the dry to the imbibed stage was associated with changes in levels of the majority of amino acids and their precursors, alcohols, sugars, organic acids and fatty acid compounds (Fig. 2, Supplemental Table 2). In the dry stage we observed higher levels of many metabolites, including organic acids, sugars, and levels of alcohols such as alpha-hydroxybutyrate, as compared to 6 h imbibed seeds (Fig. 2, Supplemental Table 2). Most prominent were oxalic acid, glycolate, threonate, glycerate and erthyryonic acid. Synthesis of oxalic acid is accomplished via several pathways. Glucose, acetate and some acids of the TCA cycle have been implied in oxalate synthesis (Chang and Beevers 1968). Moreover, glycolic and isocitric acids (Millerd et al. 1963a, b) and oxaloacetic acid (Chang and Beevers 1968) are known to donate carbon to oxalic acid in plants. The observed transient accumulation of oxalate in seed tissues could be associated with ureide degradation and subsequent amino acid synthesis, which is required for seed storage protein synthesis in developing seeds. It has been hypothesized that degradation of organic acids in seed development may provide the energy needed for metabolic activity in this period (Ilarslan et al. 1997).

Concentration of organic acids, namely, galactonate, glycolate, glycerate, erythronic acid, phosphoric acid, quinate and threonate, decreased dramatically upon imbibition. The levels of amino acid and their precursors were invariant between dry and 6 h imbibed seeds. The levels of alpha-hydroxybutyrate and the sugars xylofuranose and sucrose also exhibited considerable decrease upon imbibition. The levels of the TCA-cycle intermediate oxalate showed significant decrease while the other TCA-cycle metabolites declined even further on imbibition. The imbibed seed stage was associated with a general increase in concentrations of monomethyl phosphate, the organic acids, parbanic acid and pentonic acid, and the TCA-cycle intermediates citrate and fumarate. In contrast, the levels of gluconate, quinate, shikimate and succinate were significantly reduced. While the levels of most amino acids and their precursors were reduced to different extents, the levels of Gly, Asp, Asn and hypotaurine significantly increased. Similarly, the levels of most sugars declined but the levels of the sugar phosphates, glucose-6-phosphate and glycerol-3-phosphate were elevated significantly. This general observation suggests that the transition from dry to 6 h imbibed is associated with the activation of initially important metabolic processes needed for seed germination. It is also likely that germination is associated with a follow up of additional metabolic processes, which occur later during germination and therefore were not observed by our metabolic profiling. The major metabolic changes observed after 6 h of imbibition were significant reductions in the levels of the majority of different metabolites, which had accumulated in the dry seeds (Fig. 2, Supplemental Table 2). Yet, our present finding suggests that metabolism during the 6 h seed stage has an additional function, namely to render certain metabolites rapidly available to support metabolic recovery during imbibition. This implies that primary metabolites might be rapidly consumed to support the metabolic switch toward the enhancement of biosynthetic processes needed for early germination. The Asp-family pathway results in the synthesis of the essential amino acids Lys, Thr, Meth and Ile through several different branches (Galili 2011; Rosental et al. 2016). In addition, Thr is also metabolized to Gly, which is involved in plant photorespiration whereas Ile is a kinase well-documented donor metabolite that feeds the TCA cycle in plants. This general expression behavior of amino acid metabolism operates as part of a comprehensive program to suppress biosynthetic pathways in order to preserve the existing energy and stimulate catabolic pathways to generate additional energy, and exposes a significant regulatory metabolic link between the Asp-family pathway and the TCA cycle, whose biological function may have a major impact on the physiological response of plants to various abiotic stresses that cause energy deprivation (Baena-González and Sheen 2008). This general observation suggests that the transition from dry to 6 h imbibed is associated with the activation of initially important metabolic processes needed for seed germination. It is also likely that germination is associated with a follow up of additional metabolic processes, which occur later during germination and therefore were not observed by our metabolic profiling. In our study we show that early germination (imbibition) events are characterized by the efficient reactivation of metabolic pathways via the availability of key precursors as well as coordination of energy metabolism. Several conserved features are apparent in both seed stages analyzed, thus confirming a high biological relevance of these changes in the process of seed and seedling development (Rosental et al. 2014).

### Metabolites of similar function are highly correlated across the RIL population

**Fig. 3:**
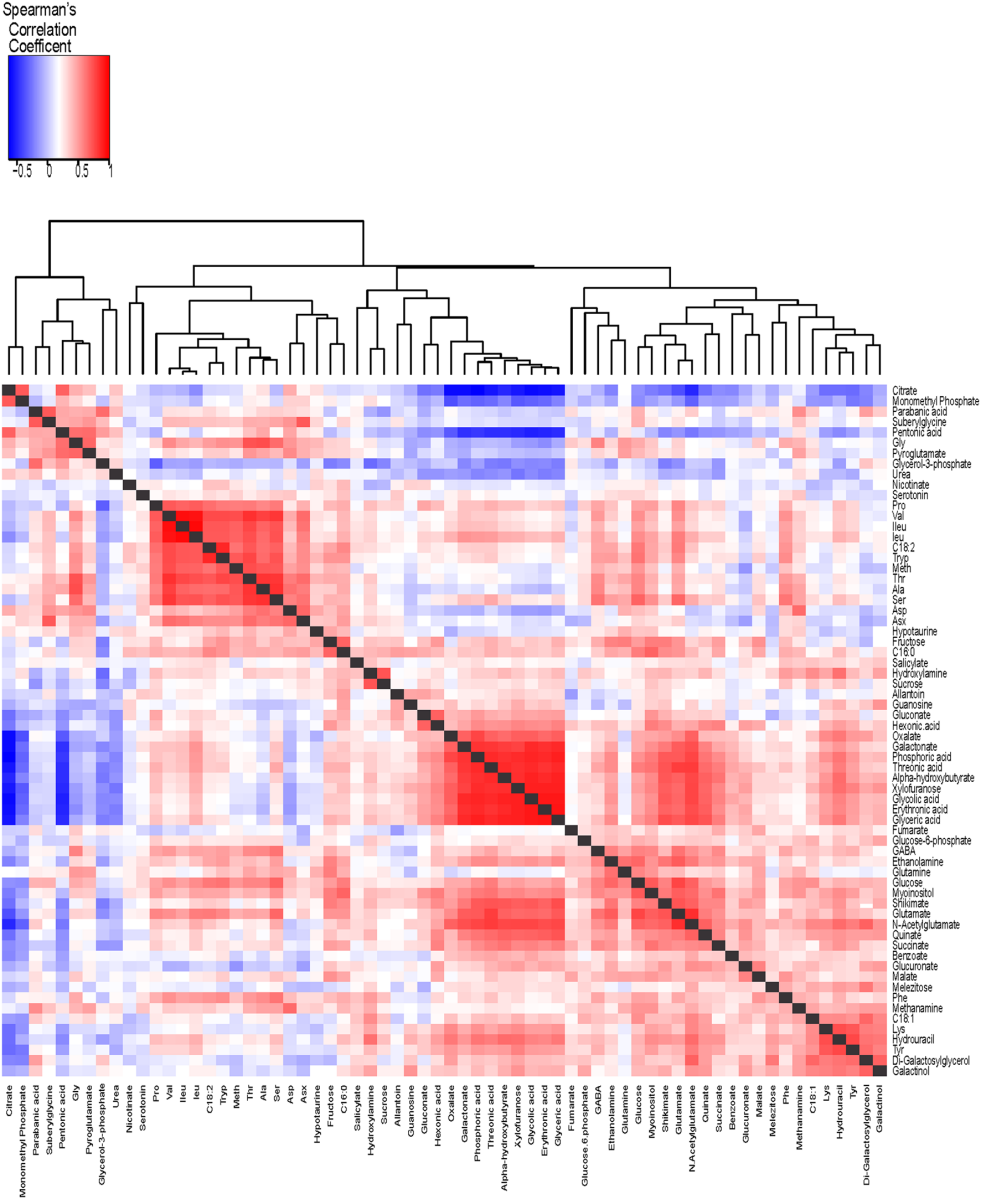
Heat map of correlations between metabolites. Each square represents the Spearman correlation coefficient between the metabolic phenotypes of the column with that of the row. Metabolic phenotype order is determined as in hierarchical clustering using the distance function 1-correlation. The dissimilarity index is employed for cluster analysis to arrange different seed phenotypes according to their similarity (Legendre and Legendre 1998). Self–self correlations are identified in black. Individual correlation coefficients can be found in Supplemental Table 4. Supplemental Fig. 3 displays the correlation heat map for all 167 metabolites found in our analysis

We created a correlation matrix of all pairwise comparisons among individual metabolites by performing Spearman rank correlation analysis for all pairs of measured traits across the whole population. Spearman’s rank correlation coefficients (Rs) and accompanying false discovery rate (FDR)-corrected P values (p_BH_; Benjamini-Hochberg) are provided in Supplemental Table 4. Unsupervised hierarchical clustering revealed several ‘‘hot spots’’ of highly correlated metabolites (Fig. 3, Supplemental Fig. 3). It is remarkable that several hot spots corresponded to the biochemical pathways to which the metabolites belong. For example, 11 of the 15 amino acids cluster in this matrix. Moreover, when we consider pairwise correlations between all amino acids, 75% had absolute correlation coefficients greater than R_s_ 0.38 (p_BH_ = 0.0001)(Supplemental Table 4). In another cluster, glycine clustered most highly with pyroglutamate (R_s_ = + 0.64; p_BH_ = 1.36E−11), but also with glycerol-3-phosphate and urea. Glucose correlated most highly with myo-inositol (R_s_ = + 0.68; p_BH_ = 9.06E−05), and GABA with glutamate (R_s_ = + 0.58; p_BH_ = 1.25E−09). Also, in the same cluster ethanolamine, glutamine, shikimate and its precursor quinate, as well as TCA intermediates malate and succinate, grouped together.

The inter-dependence of biosynthetically unrelated amino acids observed in our study concurred with that of biosynthetic related amino acids, such as Gly, Ser, Thr, Ile and Val, of which Thr, Gly, and Ile are directly associated with the Asp family (Less et al. 2010; Less and Galili 2009). Ser is closely related to Gly, and Val biosynthesis is initiated by Thr (KEGG pathway database) (Kanehisa et al. 2009). Amino acids closely related by a biochemical pathway exhibited even stronger correlations than the average in the amino acid module. The significant positive correlations between amino acids imply that ratios between amino acid levels within a seed “must” be maintained, and they reflect a highly regulated amino acid metabolism that includes both protein and non-protein amino acids (i.e. GABA), both aromatic and aliphatic, likely to occur at the post-transcriptional level in the regulation of N allocation (Toubiana et al. 2012). That said, we cannot rule out the possibility that integration of induced changes at the transcriptional level accounts for the intragenotypic correlation of amino acid metabolism. The vast number of highly significant associations between the amino acids and carbon metabolites in the seed is indicative of considerable crosstalk between C and N networks, as is exemplified by the correlation between pyruvate-nicotinate (niacin, precursor of NAD), on the one hand, and amino acids and glycolytic intermediates, on the other hand. Our results support previous suggestions of an extensively overlapping regulatory basis for central pathways in N and C metabolism (Gutiérrez et al. 2007; Nunes-Nesi et al. 2010; Stitt and Fernie 2003).

### Metabolic profiling of seeds in a tomato RIL population identifies mQTLs

**Fig. 4:**
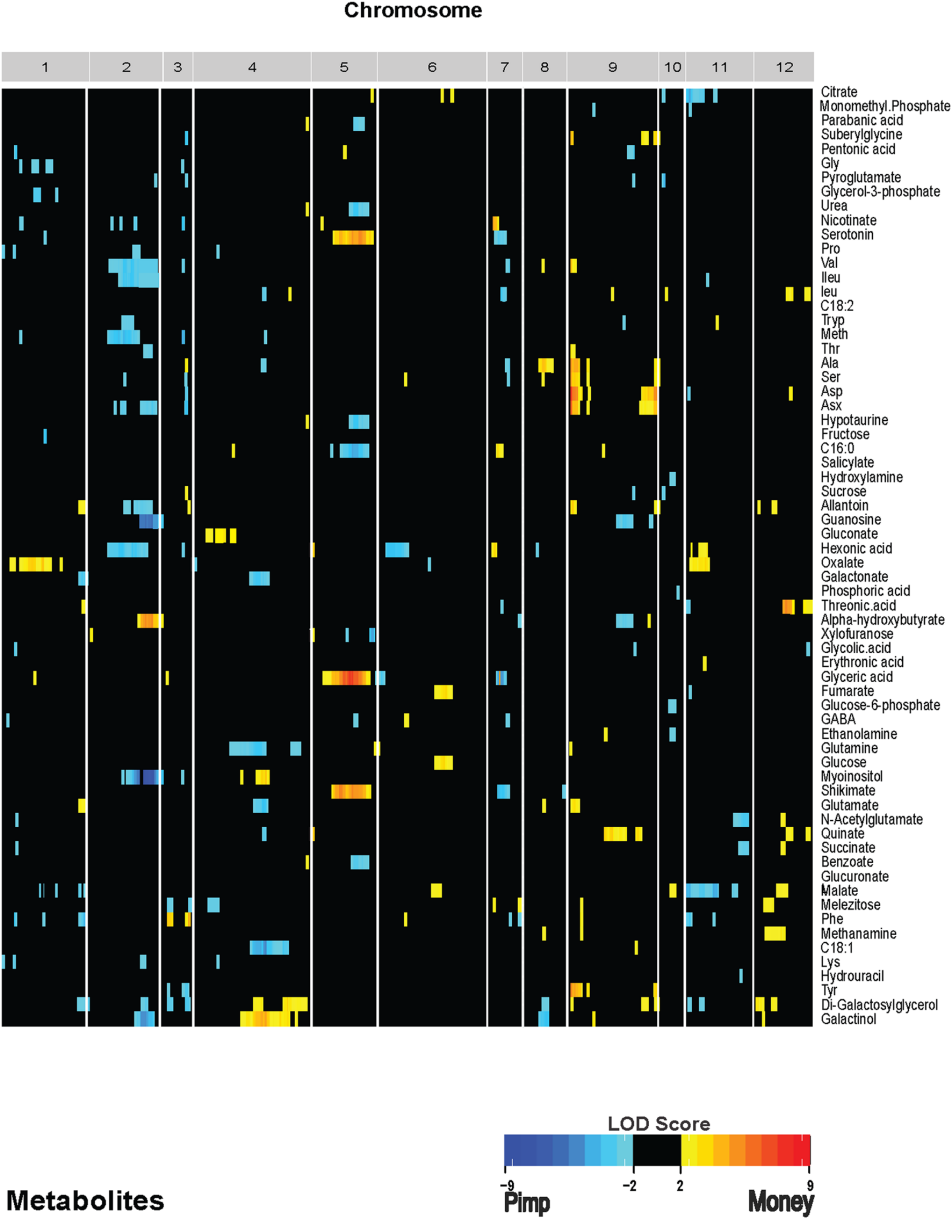

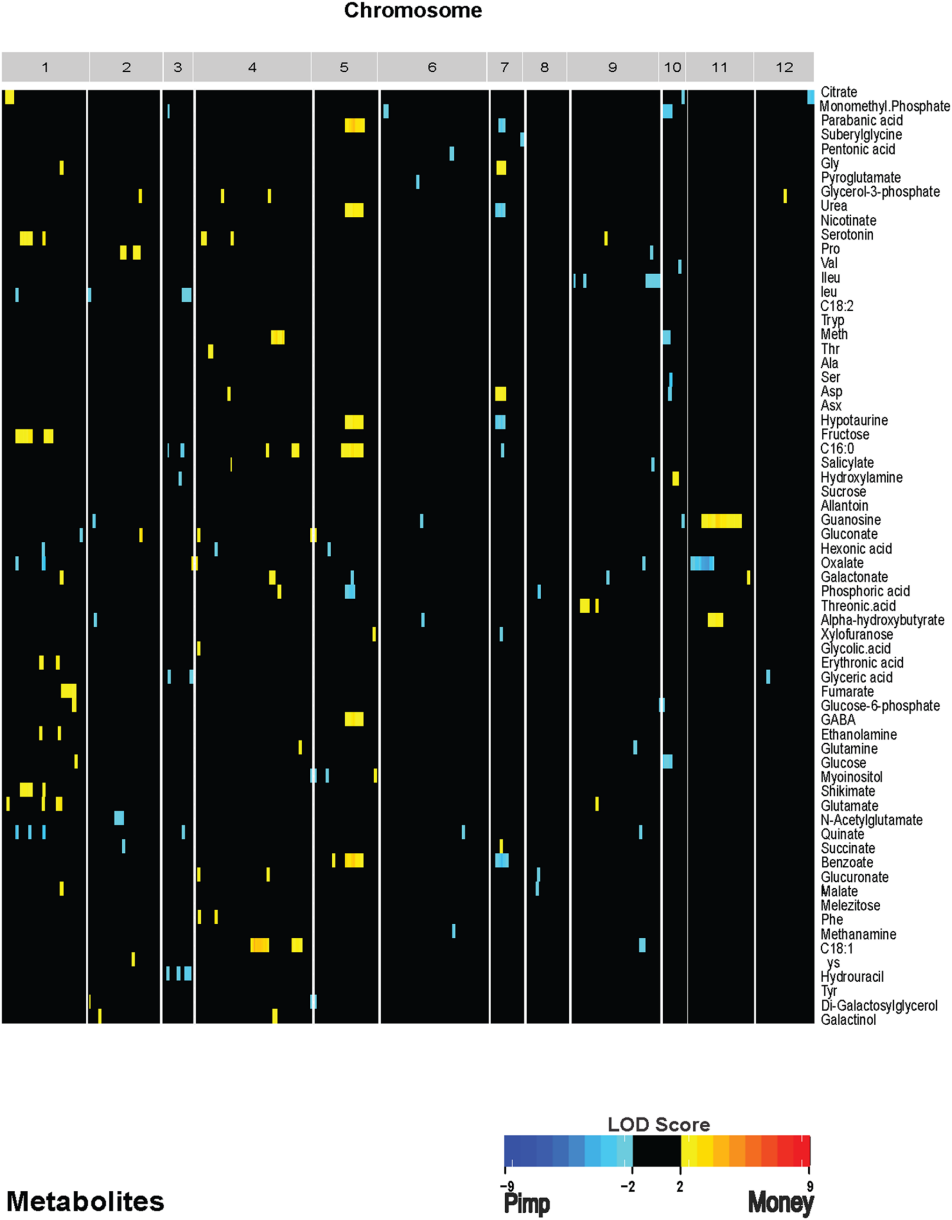
**a** Genomic locations of genetic mQTLs identified for metabolite accumulation. **b** Genomic locations of G × E mQTLs identified for metabolite accumulation. Tomato chromosomes are identified by arabic numerals (1–12), with centimorgans ascending from left to right; chromosomes are separated by white lines. Colored cells indicate QTL significant. Significant thresholds were defined with permutation analysis (n = 1000, p < 0.01) by randomizing the genotypes over each metabolite and was set to LOD > 3 accordingly. The LOD color scale is indicated, showing blue and light blue when the *Solanum pimpinellifolium* (‘Pimp’) allele, and yellow and red when the *S. lycopersicum* (Money ‘MM’) allele, at that marker results in an elevated level of metabolic phenotype. Supplemental Fig. 4a, b shows genomic locations of genetic mQTLs identified for all 167 metabolites

The purpose of the current study was to explore the possibility that the levels of metabolites in tissues are sufficiently heritable in an F8 population to provide significant linkage signals, leading to metabolic QTLs. Given that many pathways converge upon common metabolites and that these pathways have multiple controllers, any single genetic locus may not alter metabolite levels significantly, and therefore may not be identified as a metabolite QTL. Nonetheless, in our study, we found significant linkage signals, including some that are quite strong (Fig. 4, Supplemental Fig. 4). In our experimental set-up the environmental variation is defined as variation observed between the two developmental stages (dry and 6 h). A large fraction of the observed variation is due to genetic effects among concentrations of metabolites, although we also found metabolites with variation in the genetic × environmental component (Fig. 4b, Supplemental Fig. 4b). Co-location of the QTLs was expected, as there was strong correlation among metabolites, which is an indication of possibly shared mQTLs. Four of the total of eleven amino acids that clustered based on correlation (Ala, Phe were the exceptions) map to a similar position on chromosome 9 (Fig. 4, Supplemental Fig. 4). Similarly, glutamate and GABA have a QTL profile with a shared mQTL on chromosome 4. The foregoing results demonstrate that metabolites of a functional class often are correlated with one another and have common mQTLs.

Our results reveal that metabolites can be mapped to distinct genetic regions, much like mRNA transcripts. Although QTL mapping in an F_8_ population does not provide sufficient resolution to identify individual genes with high certainty, it can yield novel information about regulatory networks. Phenotypes mapping to the same locus can be hypothesized to be co-regulated by that locus. With our definition of “phenotype” including metabolites and physiological traits, we can begin to devise relationships between these phenotypes and genetic regions.

### Integration of metabolic and seed phenotypic traits

Using an analogous approach to that taken previously for metabolites (Buscher et al. 2009; Lisec et al. 2008; Meyer et al. 2007; Sulpice et al. 2010), we were also interested in unravelling possible links between previously studied seed performance phenotypes and a specific combination of metabolites. The first 20 metabolites with significant correlations under oxidative stress condition (G_max_) are listed in Supplemental Table 7 as an example, whereas details concerning connection between metabolites and different germination traits are provided in Supplemental Table 5. The highest absolute correlation found was for an unknown metabolite (RI_2442), which yielded a value of 0.406. Although the correlation is statistically highly significant (P value of 3.35E−05), it can only explain 16.48% of the variance. Other significantly correlated compounds are allantoin, alpha-hydroxybutyrate, C16:1, fructose, gluconate, glucuronate, guanosine, hexonic acid, hypotaurine, shikimate, xylofuranose and a number of unknown metabolites (Supplemental Table 5). Their individual contribution to the explained variance ranges from 5 to 15%. In contrast to the aforementioned pairwise correlation analysis, CCA yielded a much stronger correlation of 0.60. This value corresponds to 36% of variance explained by the linear combination of metabolites, almost 1–5 times more than explained by any individual metabolite. Comparing the results obtained from a combination of different metabolites and germination traits which showed significant associations, it can be seen that strongly represented metabolites are compounds of central metabolism, such as glucose and fructose, members of the tricarboxylic acid (TCA) cycle, such as succinate, citrate and fumarate, amino acid and precursors, members of the membrane/phospholipid biosynthetic pathways, such as glycerol-3-phosphate, ethanolamine and myo-insitol, or sucrose (Supplemental Table 5). Interestingly, with regard to different germination traits (G_max,_ t_10_ ^− 1^, t_50_ ^− 1^, MGR, and AUC), in particular under stress conditions, the predominantly represented metabolites are well known for abiotic stress responses. The single most remarkable observation to emerge from the data comparison was that of those stress-related metabolites, most were ominously present in the correlation with salt- and osmotically-stressed seeds for G_max_ as well as unprecedentedly for t_10_ ^− 1^, t_50_ ^− 1^, MGR and AUC, including myo-insitol, Pro, fumarate and succinate. High temperature stress was associated with Ile, Leu and Val; again their response to abiotic and biotic stresses and heat is well known, whereas strong representation of sugars (xylofurnose, fructose), organic acids (gluconate, glucornate, hexonate, shikimate etc.) and some bases and alcohols was evident in the case of G_max_ and some additional metabolites in the case of AUC under oxidative stress conditions. The overall association of metabolites with phenotypic traits across different conditions provides an indication of possible cross-talk between different abiotic stresses (e.g. salt and osmotic). It also provides insight into the nature and consequences of the genetic variation of metabolic function and its relationship with seed performance phenotypes.

### Combination of the levels of a large number of metabolites show a close correlation with seed germination

The present study shows the levels of a large number of metabolites, rather than a few individual metabolites, show a close correlation with germination parameters. It indicates that variation in the germination parameters coincides with characteristic combinatorial changes of metabolite levels, whereas individual metabolites may fluctuate largely independently of alterations in germination. CCA provided highly-ranked clusters in which metabolites of central metabolic pathways are strongly represented. Sugars of high relevance are the three metabolic hexose intermediates: glucose, sucrose and fructose. Sugars play an important role in overriding developmental regulation of seeds at a given point in time in a given cell or tissue. Previous studies have provided correlative evidence that certain sugar levels and/or the resulting changes in osmotic values are necessary within defined tissues or cells to maintain a distinct stage of differentiation or to proceed with the developmental program. Metabolites of the TCA cycle, such as succinate and fumarate are highly ranked. Also highly ranked is myo-inositol. Other metabolites, such as glycerol-3-phosphate play a major role in membrane/phospholipid biosynthesis. Other highly-ranked metabolites are the amino acids Ala, Ile, Leu, Met, Ser, Phe, Pro, Asp, Trp, Tyr, Val, as well as the sugar alcohols myo-inositol and galactinol, and the fatty acids palmitate and linoleate and alpha-hydroxybutyrate.

Our data display the occurrence of both positive and negative correlations between metabolites and different seed quality phenotypes. These findings corroborate the ideas of (Meyer et al. 2007) who found known metabolites displaying a negative correlation to biomass. These metabolites are the aforementioned intermediates of central metabolic pathways including sucrose, glucose and the TCA cycle members succinate and fumarate, as well as the amino acids Ala, Ile, Leu, Met, Ser, Phe, Pro, Asp, Trp, Tyr and Val. Although we have found both positively and negatively correlating metabolites amongst different seed quality phenotypes, the majority of the positively-correlated metabolites is a substantial fraction of metabolites related to stress responses, such as Ala, Ile, Leu, Meth, Ser, Phe, Pro and Val, as well as some unknown metabolites. Thus, a link between the metabolites ranking high in the CCA and seed quality phenotypes is plausible because central metabolism and stress responses are of the utmost importance to seed germination, and thus, to seed quality. The observed scenario depicts the fact that positively-correlated metabolites could be an attribute to plant defense against abiotic and biotic stresses. Thus, these results suggest that higher concentrations of these metabolites coincide with better-armed plants. Another possible explanation for this is that positively correlated metabolites are positive signals regulating plant growth and the contrary would be true for negatively-correlated metabolites.

### Comparative overview of QTLs for known metabolites and germination phenotypes

Furthermore, we also show that the combined use of mQTL and phQTL, with correlations allows one to derive a network and establish data-driven hypotheses about metabolite and phenotype relationships. For example, inspection of the overlap showed that several QTLs controlling primary metabolites were co-located with different seed performance phenotypes. A comprehensive overview of all overlapping mQTLs with phQTLs observed in the RIL population for known metabolites and the chromosomal localization is shown in Supplemental Fig. 5 with the number of overlapping mQTLs per phQTL ranging from 3 to 9. This indicates that there is strong genetic regulation of the metabolic and phenotypic traits investigated in this study and also points at possible cross-talk in different seed germination condition. In further support of this hypothesis some metabolites (allantoin, pentonic acid, monomethyl phosphate, melezitose etc.) display up to two QTLs co-localizing with any of the phQTL (Supplemental Fig. 5).

### Metabolomic correlation-network modules in f_8_ based on a graph-clustering approach

**Fig. 5:**
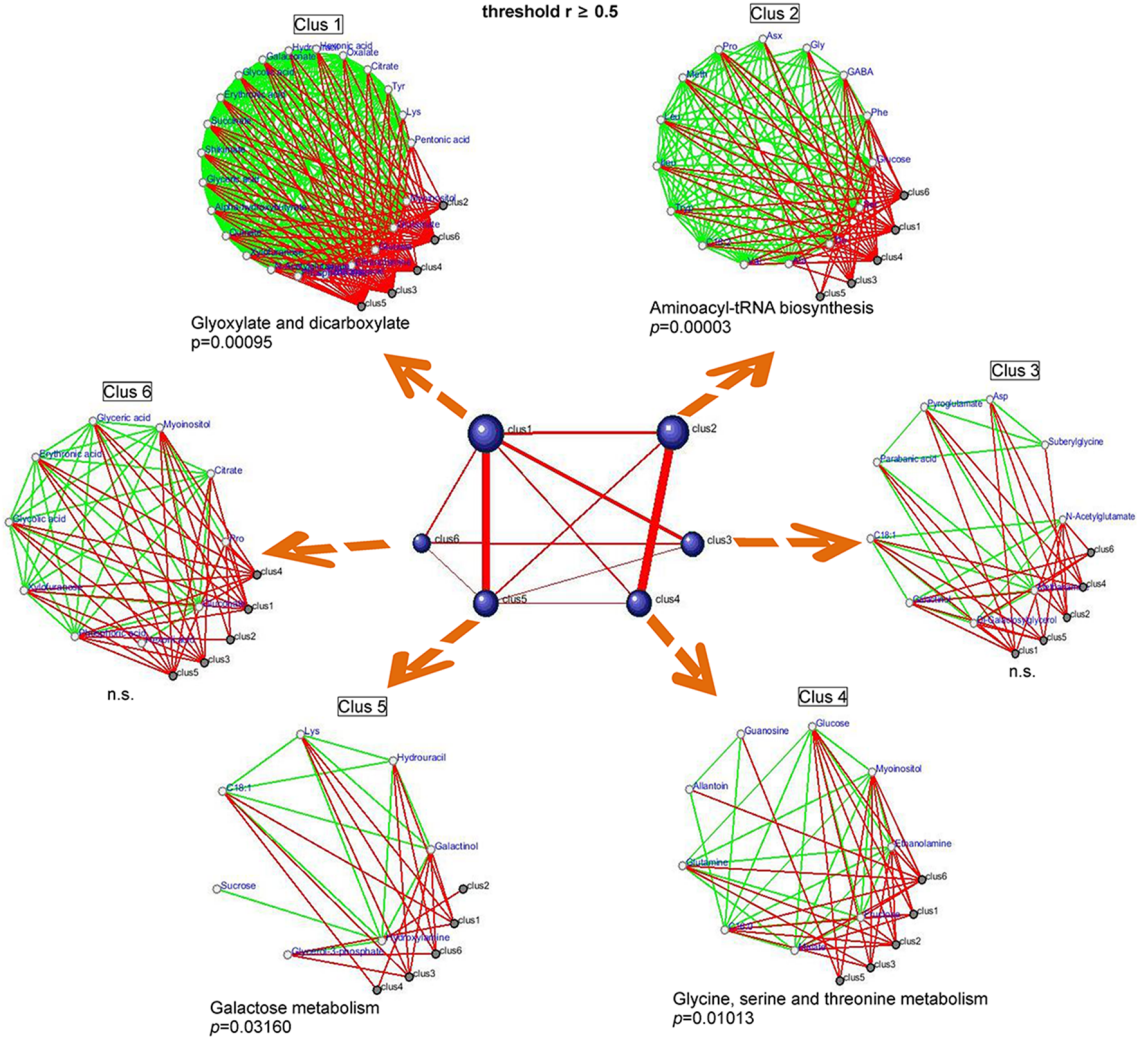
Graph clustering of correlated metabolomic modules in tomato seeds (threshold r ≥ 0.5). Using the DPClus algorithm we extracted six clusters in tomato seeds. The significant metabolic pathways were assigned by KEGG enrichment analysis (see “Methods”). The central graph consisting of six blue clusters and ten red edges was extracted by DPClus. Each blue cluster contains densely connected metabolites (see Clus1–6). Small white nodes in the clusters indicate metabolites. The internal nodes of the clusters are connected by green edges; neighboring clusters are connected by red edges

A complementary overview of the metabolomic correlations network was obtained by extracting all significant trait–trait correlations (*R*_*s*_ ≥ 0.5) and visualizing them using DPClus (Altaf-Ul-Amin et al. 2006) that identifies clusters in the metabolomic correlation network. Graph clustering using DPClus yielded densely-connected metabolites on the metabolomic correlation networks. KEGG enrichment analysis, used to assess the statistical significance of the detected clusters, demonstrated specific differences in the clusters in the enriched pathways. We postulate that the assigned KEGG pathways for each cluster reflect differences in underlying genetic properties of biochemical regulation of stage specific pathways. The largest cluster was ‘Glyoxylate and dicarboxylate metabolism’ (Fig. 5). This cluster contained metabolites associated with the biosynthetic pathways of carbohydrates from fatty acids or precursors which enter the system as acetyl-coenzyme A. Its crucial enzymes are isocitrate lyase and malate synthase and they have a relationship with several other metabolic processes: Gly, Ser, and Thr, purine metabolism, carbon fixation, ascorbate and aldarate metabolism, nitrogen metabolism, pyruvate metabolism and the citrate cycle.

Although there were no significant enriched KEGG pathways in clusters 3 and 5 (Fig. 5), these clusters may represent the extensive coordination among biosynthetic pathways involved in fatty acids biosynthesis in tomato seeds. We followed an approach that may yield new insights into the organization of metabolites in the functional pathways of a given organism (Fukushima et al. 2011). Taken together, our observations demonstrate that variations in the topology of correlation networks reflect at least partially-known biochemical pathways in tomato (Camacho et al. 2005; Fukushima et al. 2011; Steuer 2006; Toubiana et al. 2015). Our findings are in agreement with Fukushima et al. (2011) showing that graph clustering can be used to gather metabolites belonging to the metabolic pathways that change in response to different regulations. It is therefore likely that statistical KEGG enrichment analysis of such densely-connected metabolites in the correlation network is of more relevance than network similarity or proximity (Müller-Linow et al. 2007). Other studies support the idea of graph clustering approaches (Freeman et al. 2007; Fukushima et al. 2009, 2011). The aforementioned approaches have been applied effectively to gene co-expression networks for extracting functional, densely-connected genes. The present findings are consistent with a previous study which showed that the approach is also effective for metabolomic correlations (Fukushima et al. 2011).

## Conclusions

Our study shows that dry and imbibed seed developmental stages are associated with programmed metabolic switches. Specific sets of metabolic components, distributed across the metabolic network, are synthesized during seed development according to need and possible utilization of certain metabolites. These primers can be used concomitantly to predict increases in the flux of specific metabolites throughout the course of germination. Metabolite profiling in combination with significant genetic variability can reveal important regulatory mechanisms in seed metabolism and behaviour. Network analysis, coupled with our definition of “phenotype” including metabolites and physiological traits highlighted the inherent differences between developmental seed stages as well as hierarchy of regulation between physiological-related and metabolite traits. Our approach contributes to the generation of new testable hypotheses and may expand our fundamental understanding of metabolic behavior affected by genetic and/or environmental perturbations. The application of the GGG model allowed us to study the genetic basis of natural variation as well as environmental perturbations, i.e. differences between dry and imbibed seed profiles with a huge reduction in experimental load and minimal compromise in statistical power (Joosen et al. 2013a). The uniqueness of this study presents a number of important implications for future practice for the characterization of unknown gene function(s) and helps in the high-throughput screening of metabolic phenotypes (Albinsky et al. 2010; Zampieri et al. 2017). Being applied on data from heterogeneous sources, correlation-based network analysis has proven successful—from the simple understanding of topology in the metabolic correlation network through a comprehensive understanding of seed-metabolite responses to genetic alteration, to the identification of modules and metabolites with significant structural roles, which are worthy of further research (Ligterink et al. 2012). In particular, the analysis of the seed metabolic response to genetic alteration highlighted the relevance to keeping specific areas of metabolism balanced. As such, metabolic network analysis combined with genetic resources can lead to the development of significant supportive approaches in defining broader strategies for crop quality improvement. The uniqueness of this study presents a number of important implications for future practice for the characterization of unknown gene function(s) and helps in the high-throughput screening of metabolic phenotypes (Albinsky et al. 2010; Zampieri et al. 2017).

## Electronic supplementary material

Below is the link to the electronic supplementary material.

**Supplemental figure 1. Intelligent allocation of 100 RILs population to two sub-populations**. The core population of 100 RILs was divided in two subpopulations (i.e. 50 RILs for dry and 50 RILs for 6h imbibed seeds) optimized for the distribution of parental alleles using the R package DesignGG, aiming at the most accurate estimate of G and G:E effects (TIF 133 KB)

**Supplemental figure 2 A. Histogram of metabolite detection in the 100 RILs dependent upon detection in the MM and/or Pimp parental genotypes**. The axis presents the number of metabolites found in a given number of RILs (on the axis). The axis separates the metabolites into four detection classes dependent upon whether the metabolites were found in the Money ‘MM’ and /or Pimp parents. ND means that the metabolite was detected in the given parental genotype. There were 90 metabolites detected in both parents, 7 metabolites detected in only Money ‘MM’, 10 metabolites detected in only Pimp and 53 metabolites detected in neither parent for dry seeds while 76 metabolites detected in both parents, 22 metabolites detected in only Money ‘MM’, 3 metabolites detected in only Pimp and 59 metabolites detected in neither parent for 6h imbibed seeds (TIF 90 KB)

**Supplemental figure 2 B. Positive and negative transgressive segregation**. Shown are distributions of glycerol-3-phosphate (top) and Hydroxylamine (bottom) within *S. lycopersicum* × *S. pimpinellifolium* RILs as examples of positive and negative transgressive segregation, respectively. Labels ‘MM’ and ‘Pimp’ show the average accumulation of these metabolites in the parental genotypes (PDF 1811 KB)

**Supplemental figure 3**. Heat map of correlations between all 167 metabolites. Each square represents the Spearman correlation coefficient between the metabolic phenotypes of the column with that of the row. Metabolic phenotype order is determined as in hierarchical clustering using the distance function 1-correlation (TIF 3042 KB)

**Supplemental figure 4. A- Genomic locations of Genetic mQTLs identified for all 167 metabolites. B- Genomic locations of G** × **E mQTLs identified for all 167 metabolites**. Tomato chromosomes are identified by arabic numerals (1–12), with centimorgans ascending from left to right; chromosomes are separated by white lines. Colored cells indicate QTL significant. Significant thresholds were defined with permutation analysis (n = 1000, p < 0.01) by randomizing the genotypes over each metabolite and was set to LOD > 3 accordingly. The LOD color scale is indicated, showing blue and light blue when the *Solanum pimpinellifolium* (‘Pimp’) allele, and yellow and red when the *Solanum lycopersicum* (Money ‘MM’) allele, at that marker results in an elevated level of metabolic phenotype (TIF 195 KB)

Supplementary material 6 (TIF 202 KB)

**Supplemental figure 5. Overview of distribution of overlapping metabolic and phenotypic QTLs**. See legend to Supplemental figure 4A and 4B for description (TIF 394 KB)

**Supplemental figure 6. Correlation network properties of the 66 metabolites in the tomato seeds across a range of correlation coefficients**. Networks were constructed for a range of correlation thresholds from 0 to 1.0 by 0.01 increments, and each resulting network was calculated for: (A) the graph density - the ratio of the number of edges and the number of possible edges, (B) the clustering coefficient, (C) the average degree of all nodes, (D) the average path length, (E) the number of connected components, and (F) the number of metabolite-metabolite correlations (edges) in the network. Within each plot, black solid circles represent the observed data points; black dots represent 100 randomized data (TIF 280 KB)

**Supplemental table 1. List of nutrients with their concentrations which were used as fertilizer for the tomato plants** (XLSX 41 KB)

**Supplemental table 2A. List of analyzed known and unknown metabolites and their distribution among the two parents and RIL lines of the studied RIL population**. Abundancies are based on the quantification mass. Known metabolites are matched against several libraries. RI, MS match scores and probability are noted for each identified metabolite. **2B. Level of identification according to L.W. Sumner et al. 2007 and category for known metabolites** (XLSX 325 KB)

**Supplemental table 3. EI fragments of each known and unknown metabolite that are used for identification of compounds** (XLSX 10 KB)

**Supplemental table 4. Spearman R**_**s**_ **values and associated P Values for all pairwise correlations between known metabolites (Supplemental table 4A) and between all 167 metabolites (Supplemental table 4B)** (XLSX 11 KB)

**Supplemental table 5A. List of significantly correlated metabolites resulting from pairwise correlations (ordered by correlation). Supplemental table 5B. List of all relevant metabolites determined by the correlation between them and the canonical variate (ordered by absolute correlation) and ranked according to the strength of the canonical correlation** (XLSX 41 KB)

**Supplemental table 6. List of KEGG pathways used in this study** (XLSX 1243 KB)

**Supplemental table 7. List of top 20 signature metabolites ranked according to the strength of the canonical correlation (G**_**max**_ **Oxidative Stress; 300mM H**_**2**_ **O**_**2**_ **)** (DOCX 13 KB)
