# Supplementary figures and images for: Metabolomic analysis of tomato seed germination
Source: Metabolomics. 2017 Oct 23;13(12):145. doi: 10.1007/s11306-017-1284-x (PMC5653705; doi:10.1007/s11306-017-1284-x)

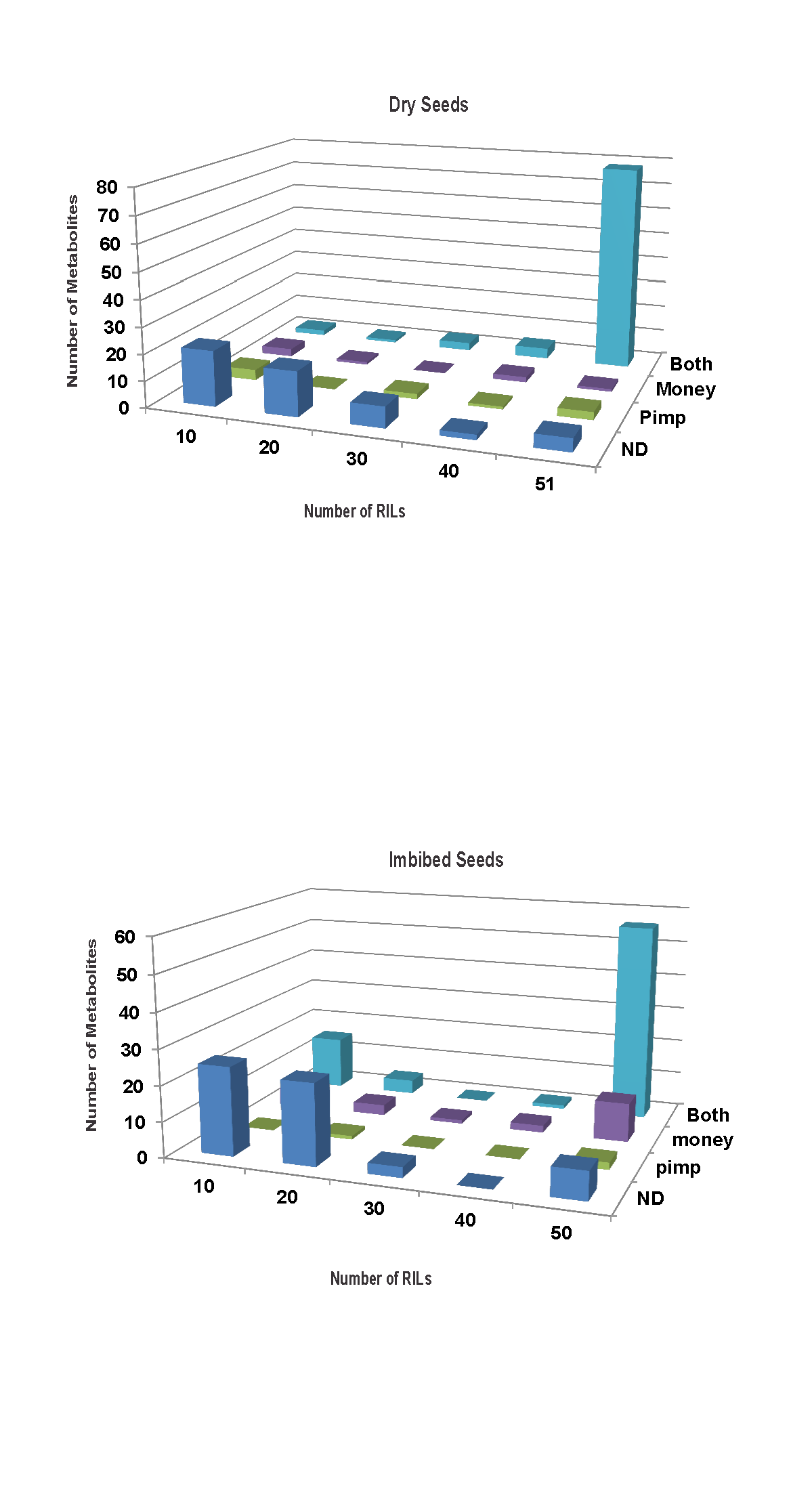

Supplement: Supplementary file 1 — Supplemental figure 1. Intelligent allocation of 100 RILs population to two sub-populations. The core population of 100 RILs was divided in two subpopulations (i.e. 50 RILs for dry and 50 RILs for 6h imbibed seeds) optimized for the distribution of parental alleles using the R package DesignGG, aiming at the most accurate estimate of G and G:E effects (TIF 133 KB) [file 11306_2017_1284_MOESM1_ESM.tif]

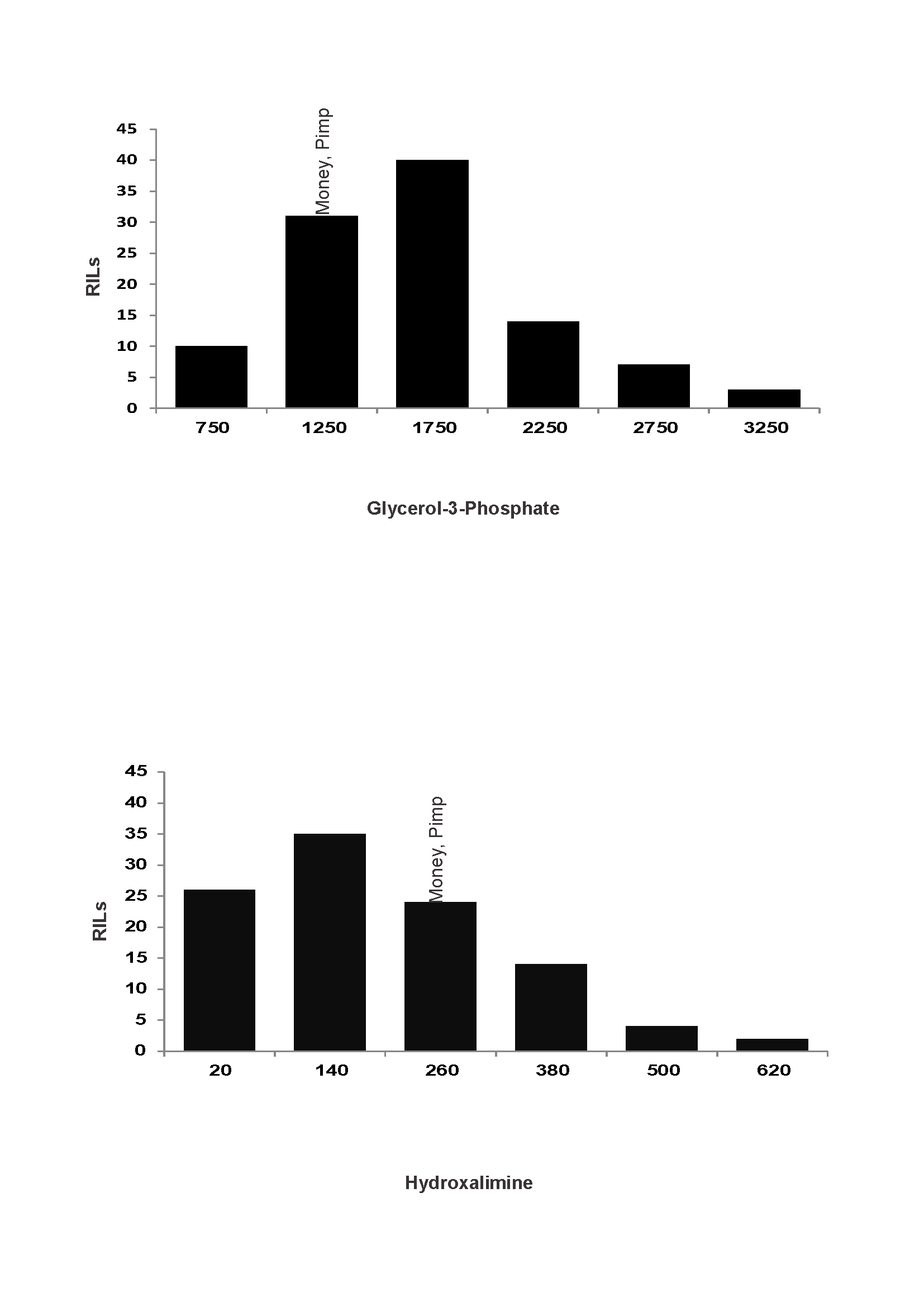

Supplement: Supplementary file 2 — Supplemental figure 2 A. Histogram of metabolite detection in the 100 RILs dependent upon detection in the MM and/or Pimp parental genotypes. The axis presents the number of metabolites found in a given number of RILs (on the axis). The axis separates the metabolites into four detection classes dependent upon whether the metabolites were found in the Money ‘MM’ and /or Pimp parents. ND means that the metabolite was detected in the given parental genotype. There were 90 metabolites detected in both parents, 7 metabolites detected in only Money ‘MM’, 10 metabolites detected in only Pimp and 53 metabolites detected in neither parent for dry seeds while 76 metabolites detected in both parents, 22 metabolites detected in only Money ‘MM’, 3 metabolites detected in only Pimp and 59 metabolites detected in neither parent for 6h imbibed seeds (TIF 90 KB) [file 11306_2017_1284_MOESM2_ESM.tif]

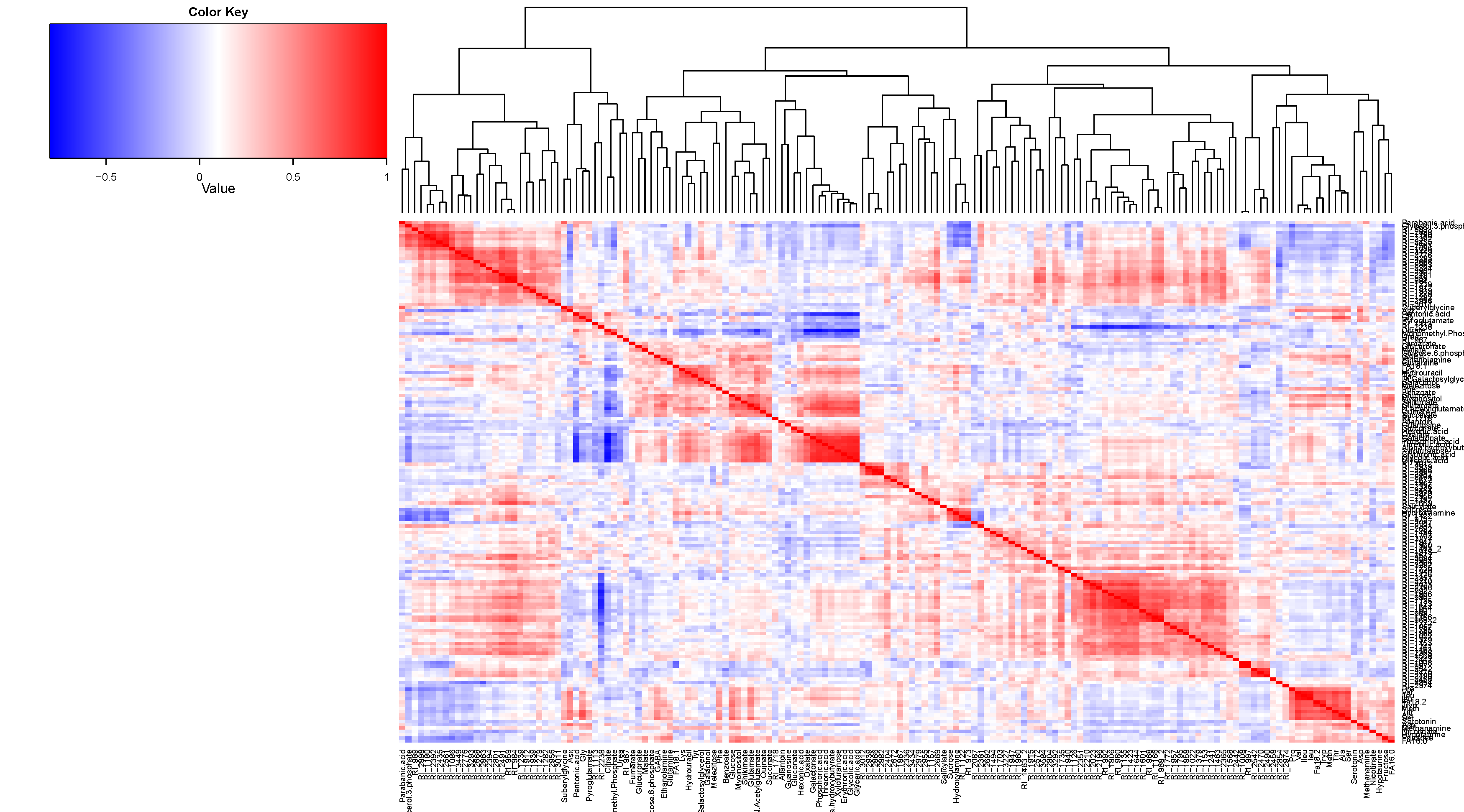

Supplement: Supplementary file 4 — Supplemental figure 3. Heat map of correlations between all 167 metabolites. Each square represents the Spearman correlation coefficient between the metabolic phenotypes of the column with that of the row. Metabolic phenotype order is determined as in hierarchical clustering using the distance function 1-correlation (TIF 3042 KB) [file 11306_2017_1284_MOESM4_ESM.tif]

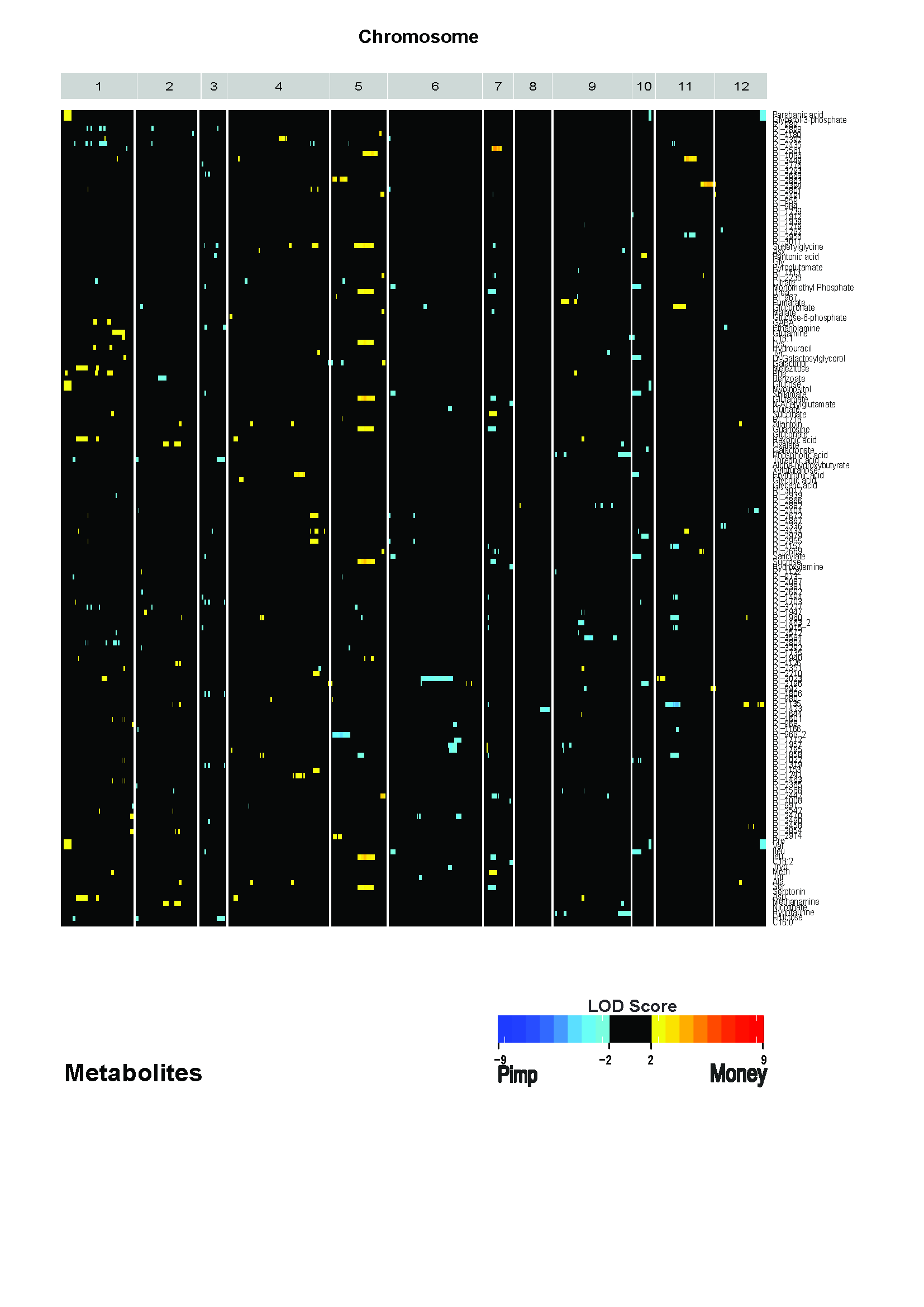

Supplement: Supplementary file 5 — Supplemental figure 4. A- Genomic locations of Genetic mQTLs identified for all 167 metabolites. B- Genomic locations of G × E mQTLs identified for all 167 metabolites. Tomato chromosomes are identified by arabic numerals (1–12), with centimorgans ascending from left to right; chromosomes are separated by white lines. Colored cells indicate QTL significant. Significant thresholds were defined with permutation analysis (n = 1000, p < 0.01) by randomizing the genotypes over each metabolite and was set to LOD > 3 accordingly. The LOD color scale is indicated, showing blue and light blue when the Solanum pimpinellifolium (‘Pimp’) allele, and yellow and red when the Solanum lycopersicum (Money ‘MM’) allele, at that marker results in an elevated level of metabolic phenotype (TIF 195 KB) [file 11306_2017_1284_MOESM5_ESM.tif]

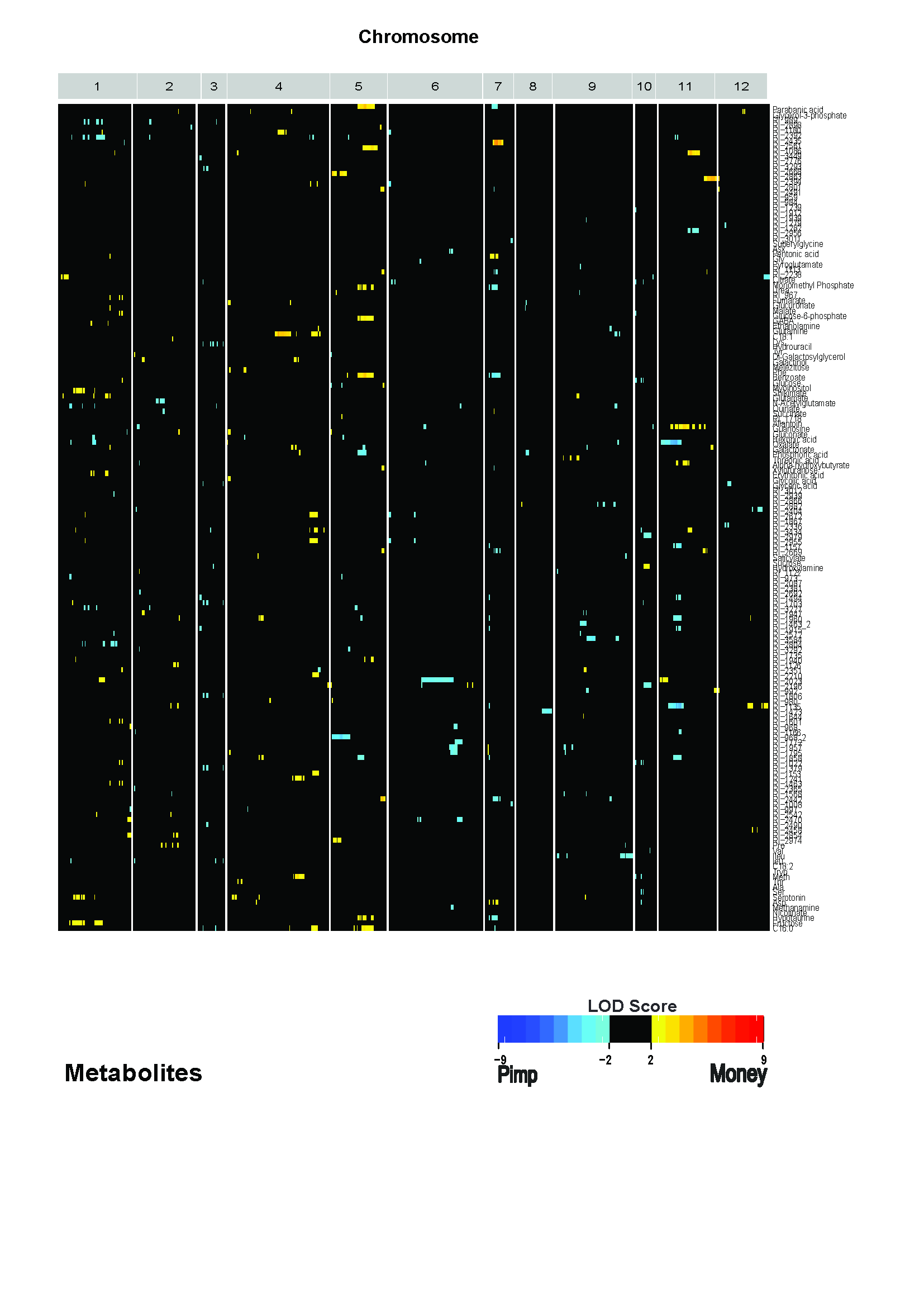

Supplement: Supplementary file 6 — Supplementary material 6 (TIF 202 KB) [file 11306_2017_1284_MOESM6_ESM.tif]

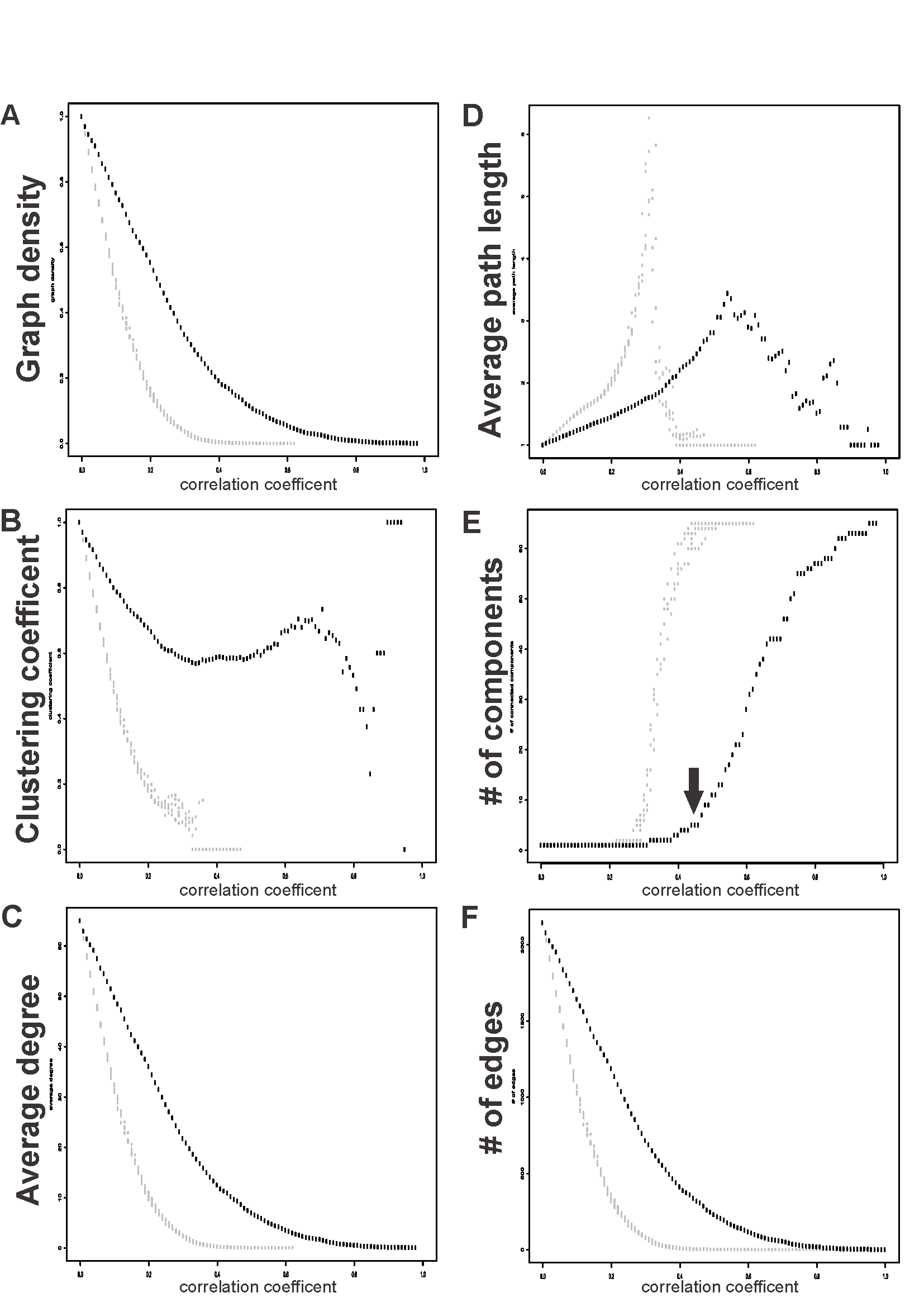

Supplement: Supplementary file 7 — Supplemental figure 5. Overview of distribution of overlapping metabolic and phenotypic QTLs. See legend to Supplemental figure 4A and 4B for description (TIF 394 KB) [file 11306_2017_1284_MOESM7_ESM.tif]

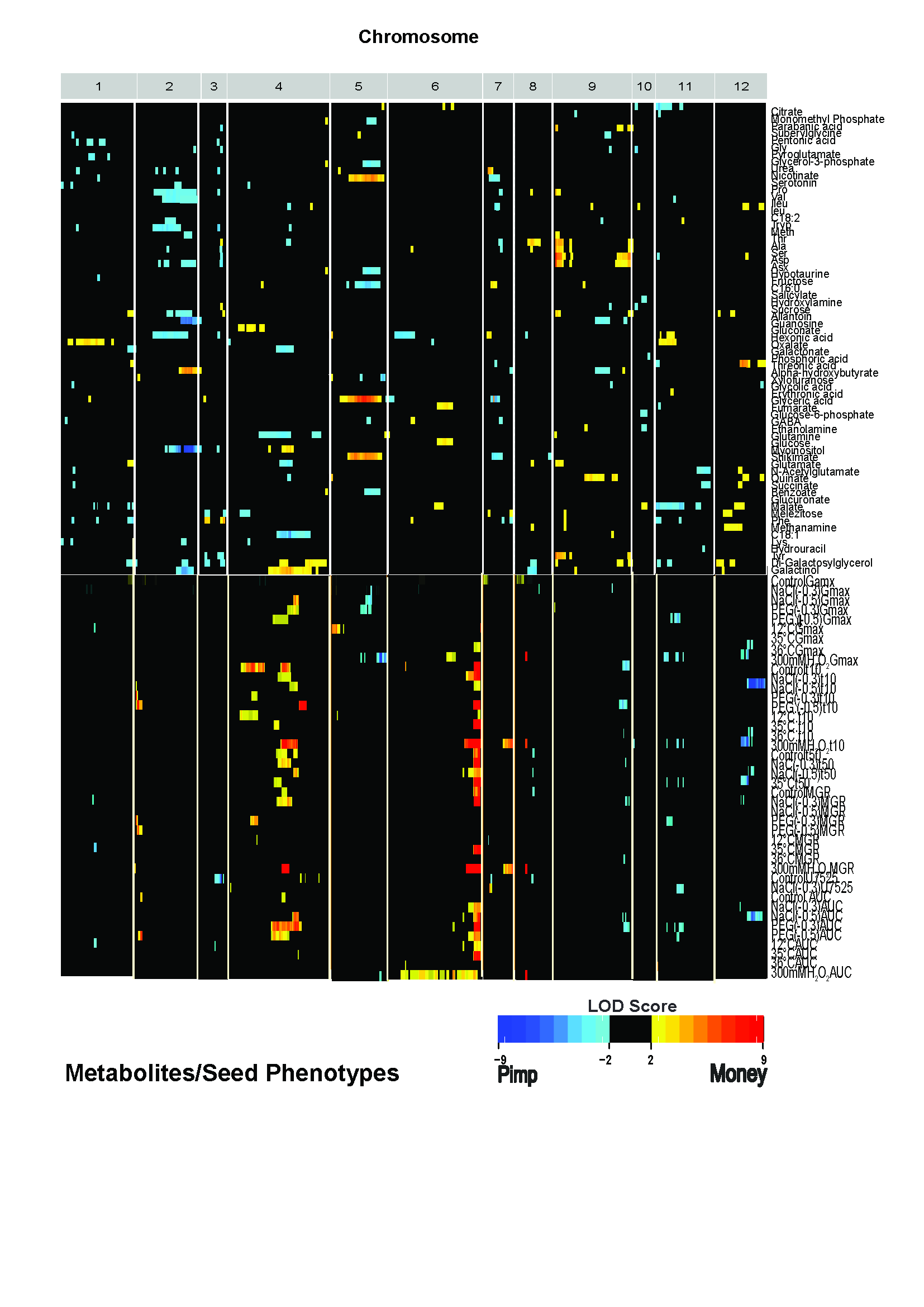

Supplement: Supplementary file 8 — Supplemental figure 6. Correlation network properties of the 66 metabolites in the tomato seeds across a range of correlation coefficients. Networks were constructed for a range of correlation thresholds from 0 to 1.0 by 0.01 increments, and each resulting network was calculated for: (A) the graph density - the ratio of the number of edges and the number of possible edges, (B) the clustering coefficient, (C) the average degree of all nodes, (D) the average path length, (E) the number of connected components, and (F) the number of metabolite-metabolite correlations (edges) in the network. Within each plot, black solid circles represent the observed data points; black dots represent 100 randomized data (TIF 280 KB) [file 11306_2017_1284_MOESM8_ESM.tif]
