# Supplementary material for: Metabolomic analysis of tomato seed germination
Source: Metabolomics. 2017 Oct 23;13(12):145. doi: 10.1007/s11306-017-1284-x (PMC5653705; doi:10.1007/s11306-017-1284-x)
Supplement: Supplementary file 3 — Supplemental figure 2 B. Positive and negative transgressive segregation. Shown are distributions of glycerol-3-phosphate (top) and Hydroxylamine (bottom) within S. lycopersicum × S. pimpinellifolium RILs as examples of positive and negative transgressive segregation, respectively. Labels ‘MM’ and ‘Pimp’ show the average accumulation of these metabolites in the parental genotypes (PDF 1811 KB) [file 11306_2017_1284_MOESM3_ESM.pdf]

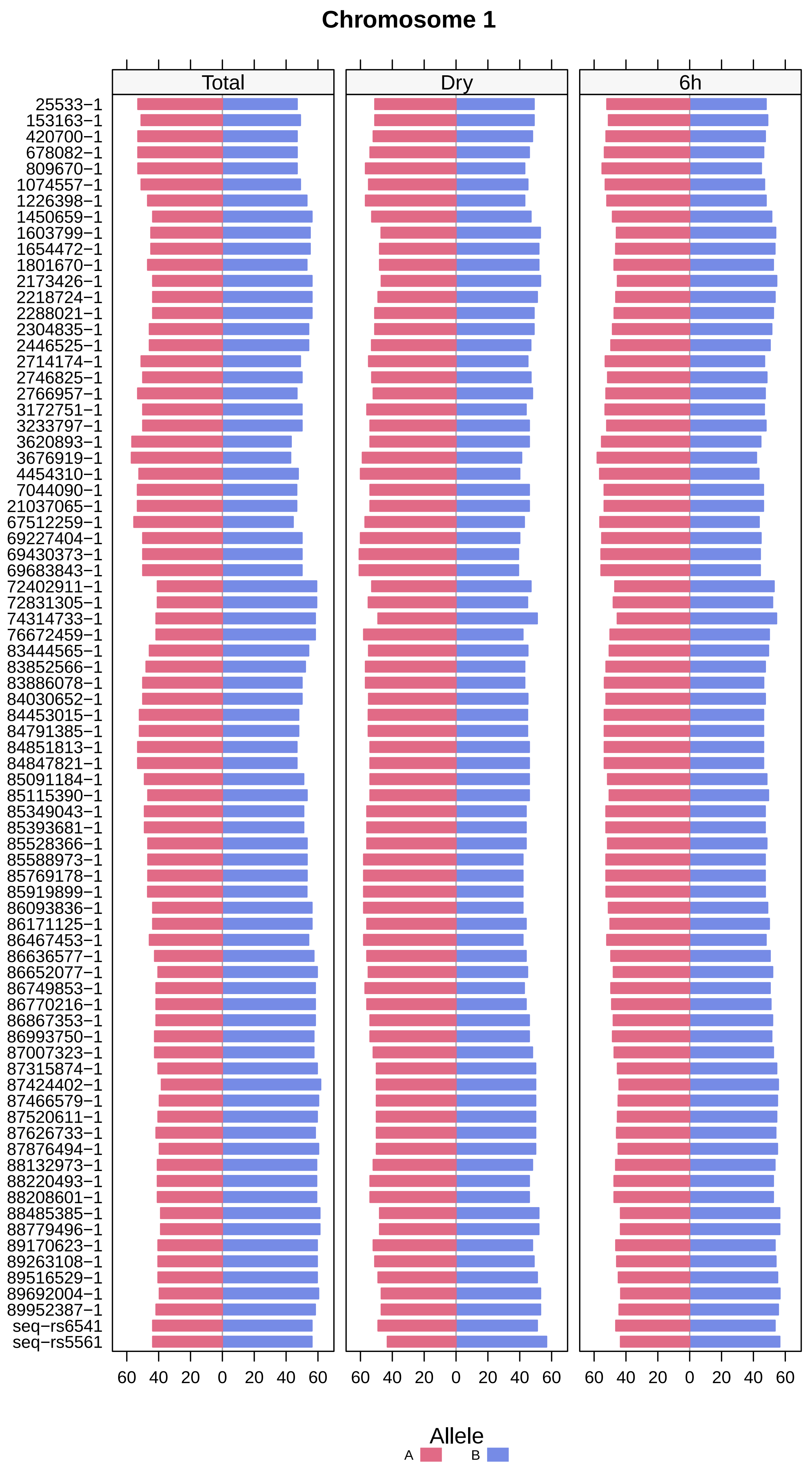
Chromosome 1
Total
Dry
6h
25533−1
153163−1
420700−1
678082−1
809670−1
1074557−1
1226398−1
1450659−1
1603799−1
1654472−1
1801670−1
2173426−1
2218724−1
2288021−1
2304835−1
2446525−1
2714174−1
2746825−1
2766957−1
3172751−1
3233797−1
3620893−1
3676919−1
4454310−1
7044090−1
21037065−1
67512259−1
69227404−1
69430373−1
69683843−1
72402911−1
72831305−1
74314733−1
76672459−1
83444565−1
83852566−1
83886078−1
84030652−1
84453015−1
84791385−1
84851813−1
84847821−1
85091184−1
85115390−1
85349043−1
85393681−1
85528366−1
85588973−1
85769178−1
85919899−1
86093836−1
86171125−1
86467453−1
86636577−1
86652077−1
86749853−1
86770216−1
86867353−1
86993750−1
87007323−1
87315874−1
87424402−1
87466579−1
87520611−1
87626733−1
87876494−1
88132973−1
88220493−1
88208601−1
88485385−1
88779496−1
89170623−1
89263108−1
89516529−1
89692004−1
89952387−1
seq−rs6541
seq−rs5561
60 40 20 0 20 40 60
Allele
A
B

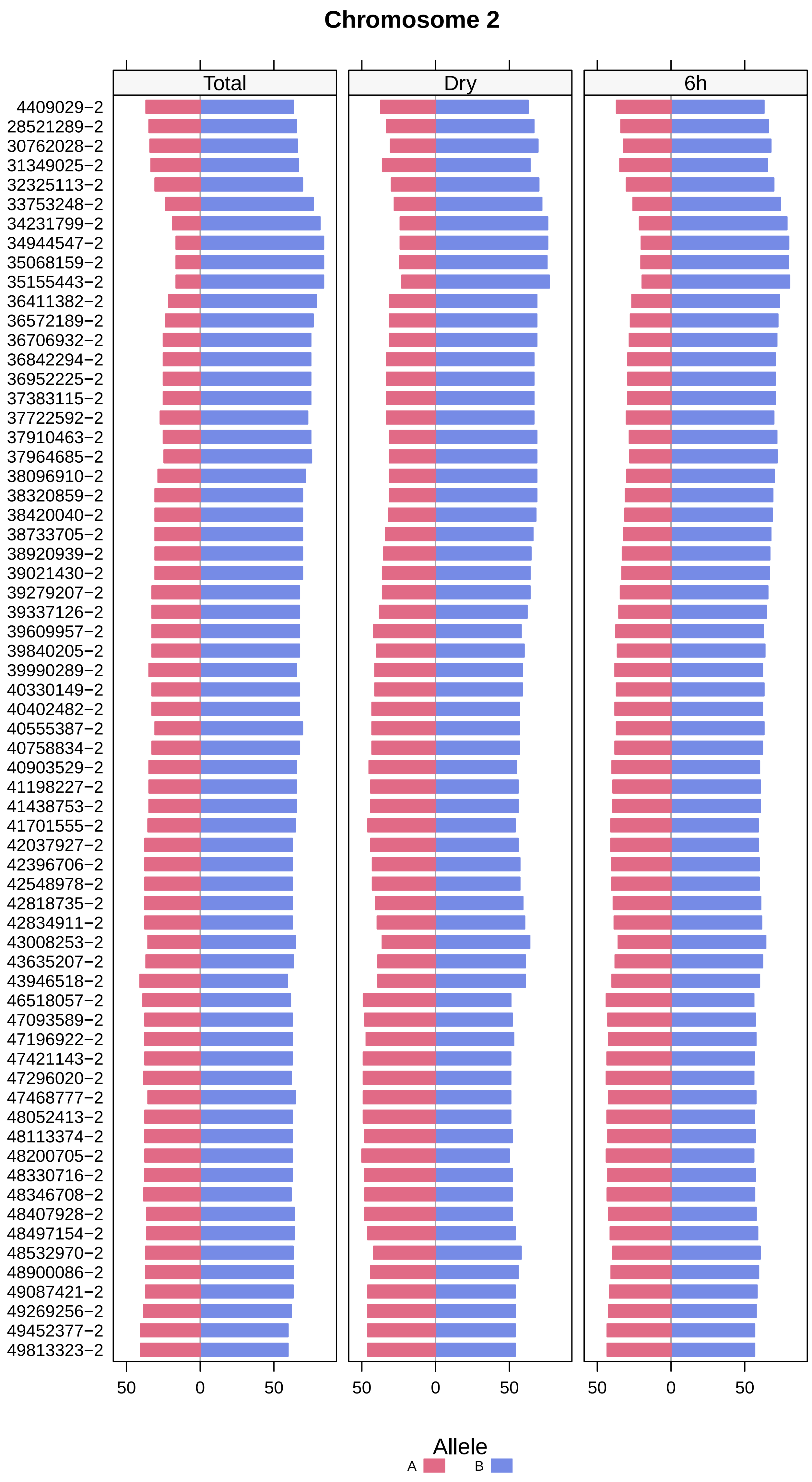

Chromosome 2
Total
Dry
6h
4409029−2
28521289−2
30762028−2
31349025−2
32325113−2
33753248−2
34231799−2
34944547−2
35068159−2
35155443−2
36411382−2
36572189−2
36706932−2
36842294−2
36952225−2
37383115−2
37722592−2
37910463−2
37964685−2
38096910−2
38320859−2
38420040−2
38733705−2
38920939−2
39021430−2
39279207−2
39337126−2
39609957−2
39840205−2
39990289−2
40330149−2
40402482−2
40555387−2
40758834−2
40903529−2
41198227−2
41438753−2
41701555−2
42037927−2
42396706−2
42548978−2
42818735−2
42834911−2
43008253−2
43635207−2
43946518−2
46518057−2
47093589−2
47196922−2
47421143−2
47296020−2
47468777−2
48052413−2
48113374−2
48200705−2
48330716−2
48346708−2
48407928−2
48497154−2
48532970−2
48900086−2
49087421−2
49269256−2
49452377−2
49813323−2
50
0
50
Allele
A
B

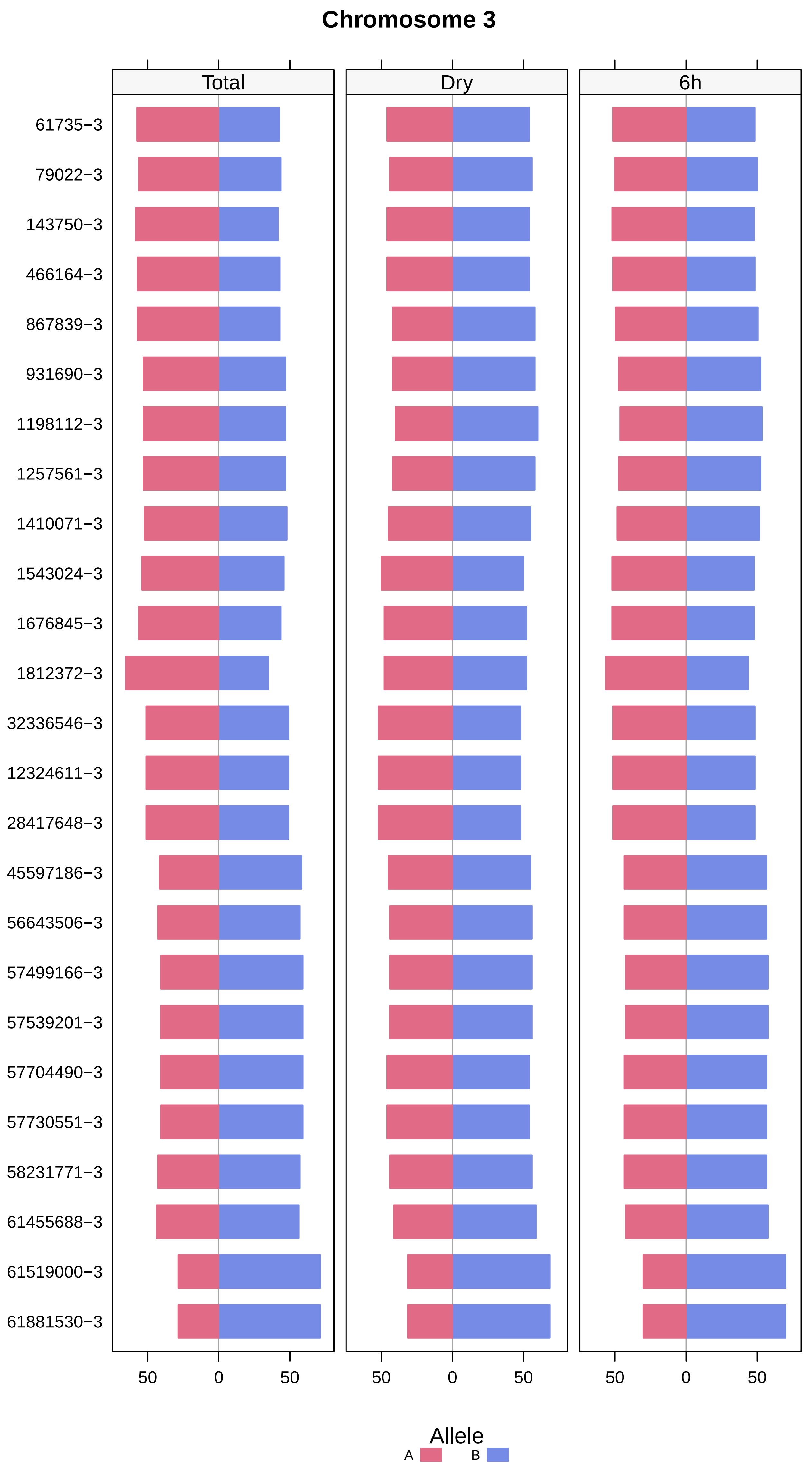

Chromosome 3
Total
Dry
6h
61735−3
79022−3
143750−3
466164−3
867839−3
931690−3
1198112−3
1257561−3
1410071−3
1543024−3
1676845−3
1812372−3
32336546−3
12324611−3
28417648−3
45597186−3
56643506−3
57499166−3
57539201−3
57704490−3
57730551−3
58231771−3
61455688−3
61519000−3
61881530−3
50 0 50
50 0 50
50 0 50
Allele
A
B

Chromosome 4

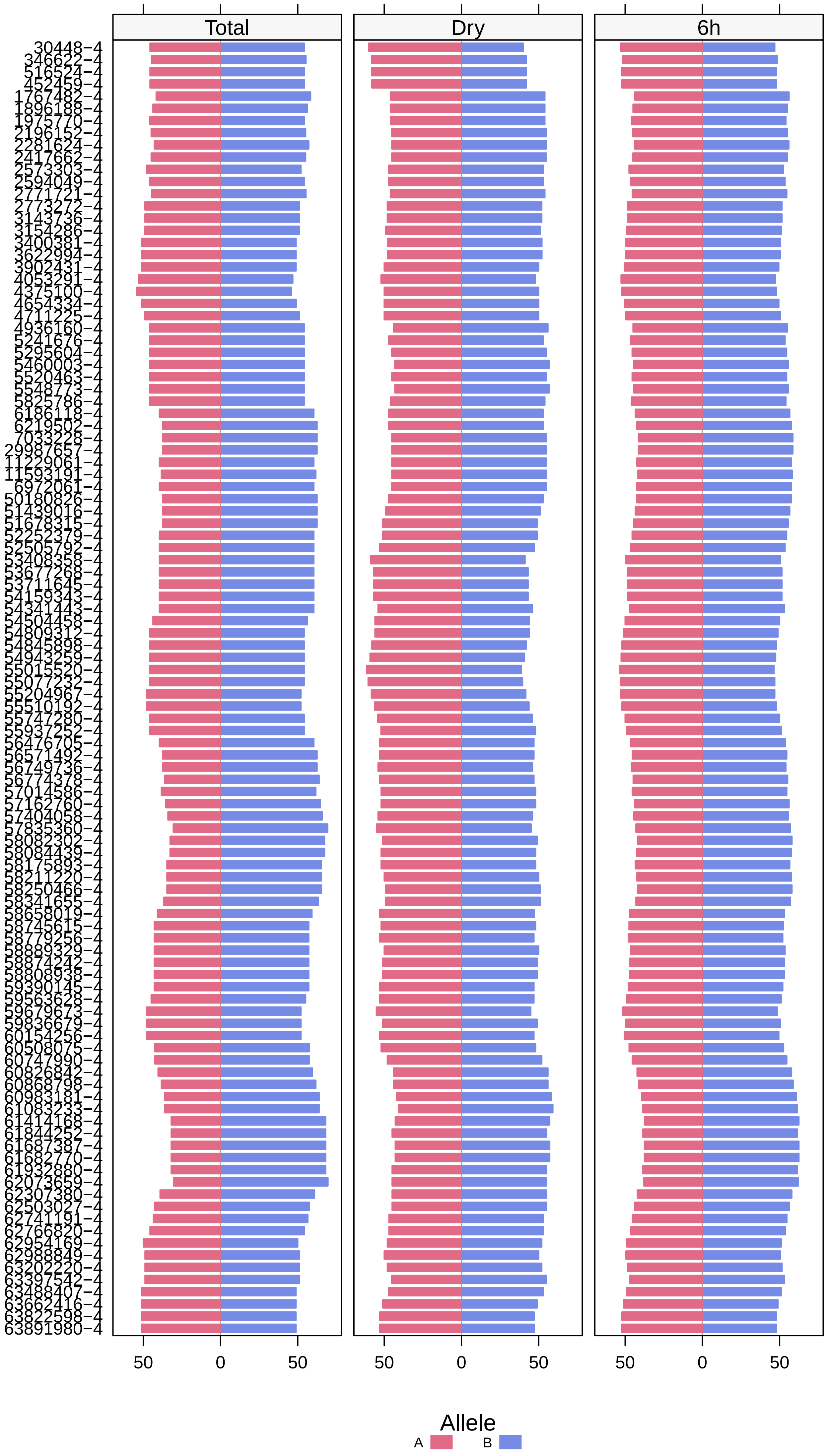

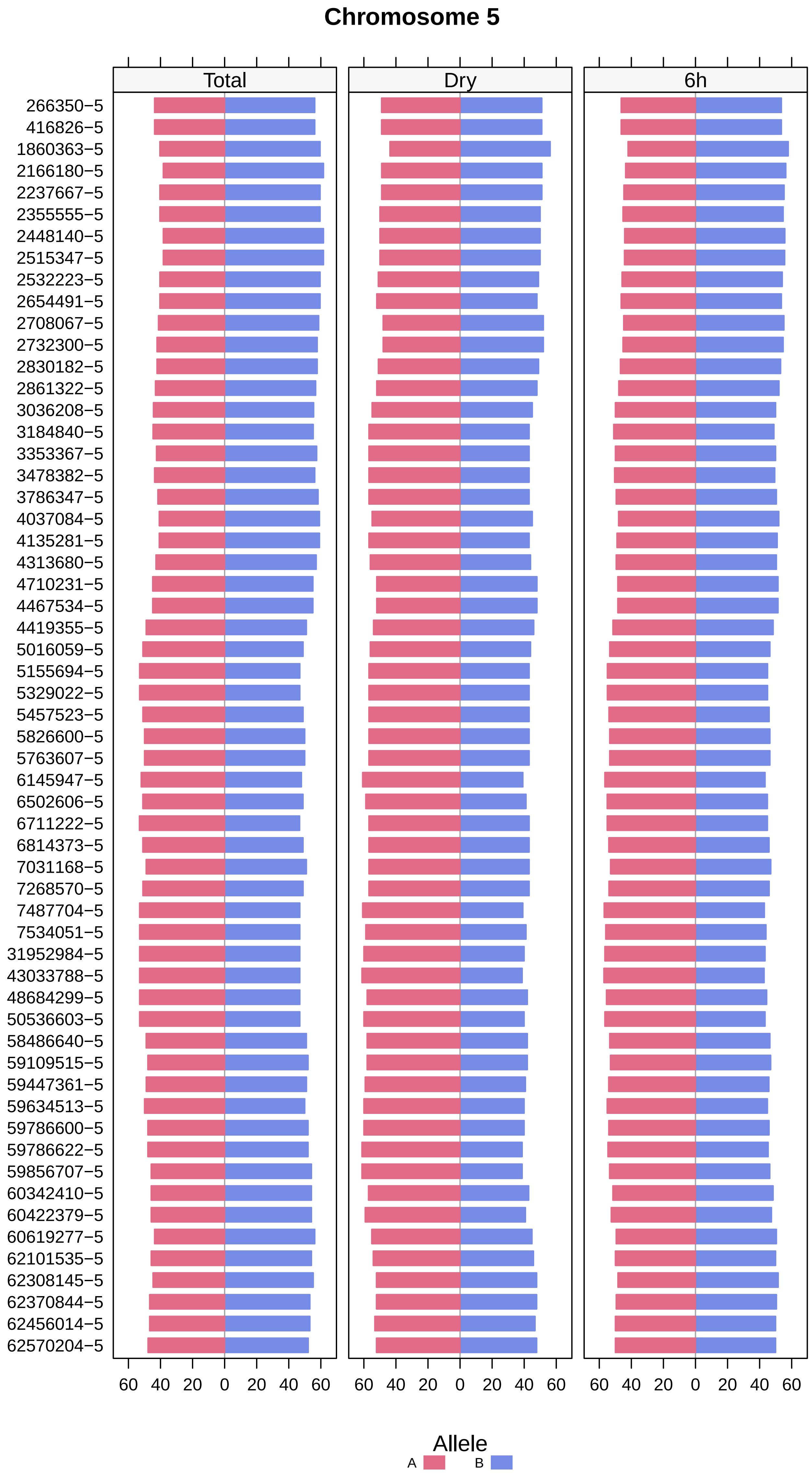

Chromosome 5
Total
Dry
6h
266350−5
416826−5
1860363−5
2166180−5
2237667−5
2355555−5
2448140−5
2515347−5
2532223−5
2654491−5
2708067−5
2732300−5
2830182−5
2861322−5
3036208−5
3184840−5
3353367−5
3478382−5
3786347−5
4037084−5
4135281−5
4313680−5
4710231−5
4467534−5
4419355−5
5016059−5
5155694−5
5329022−5
5457523−5
5826600−5
5763607−5
6145947−5
6502606−5
6711222−5
6814373−5
7031168−5
7268570−5
7487704−5
7534051−5
31952984−5
43033788−5
48684299−5
50536603−5
58486640−5
59109515−5
59447361−5
59634513−5
59786600−5
59786622−5
59856707−5
60342410−5
60422379−5
60619277−5
62101535−5
62308145−5
62370844−5
62456014−5
62570204−5
60 40 20 0 20 40 60
Allele
A
B

Chromosome 6

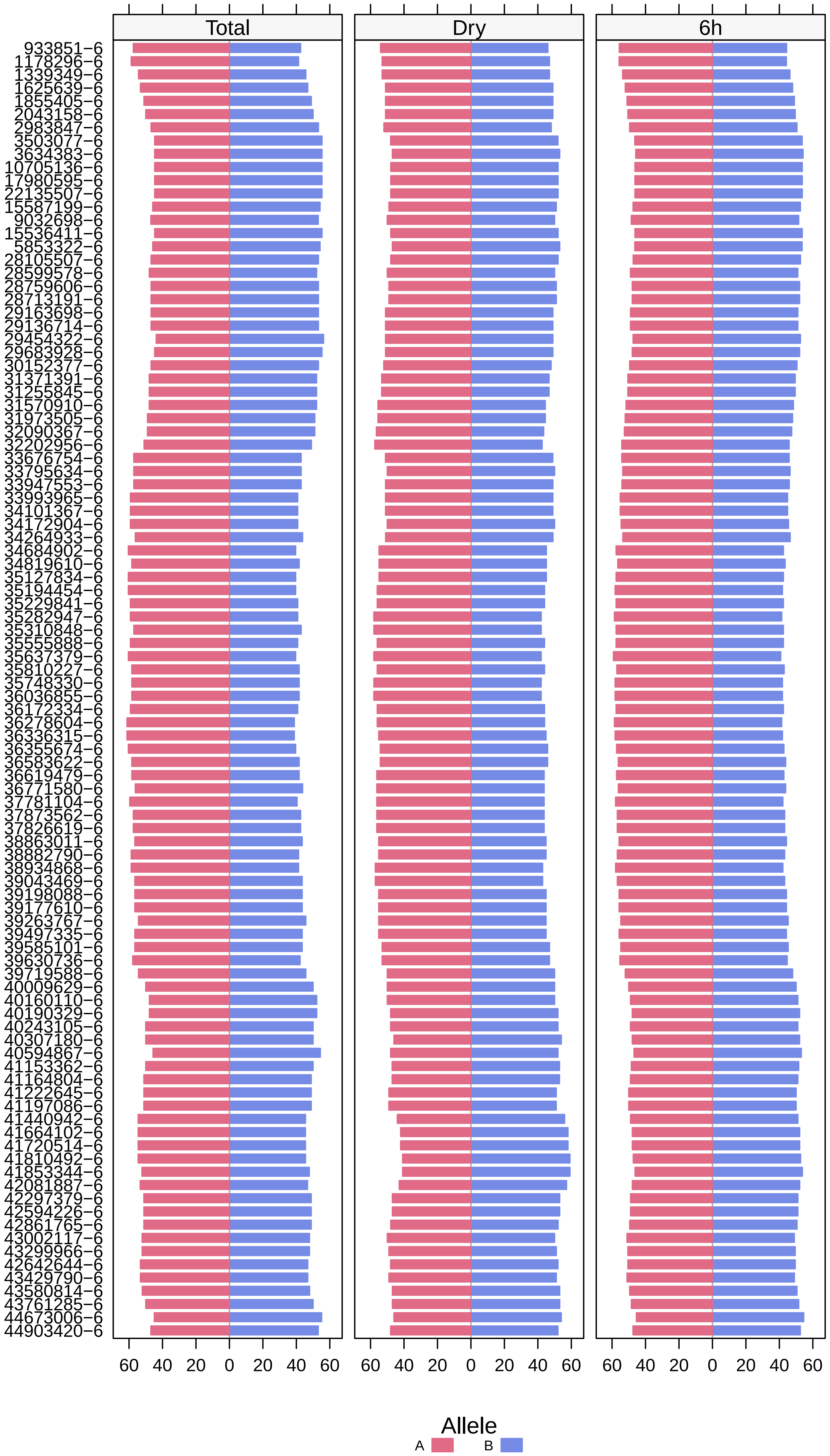

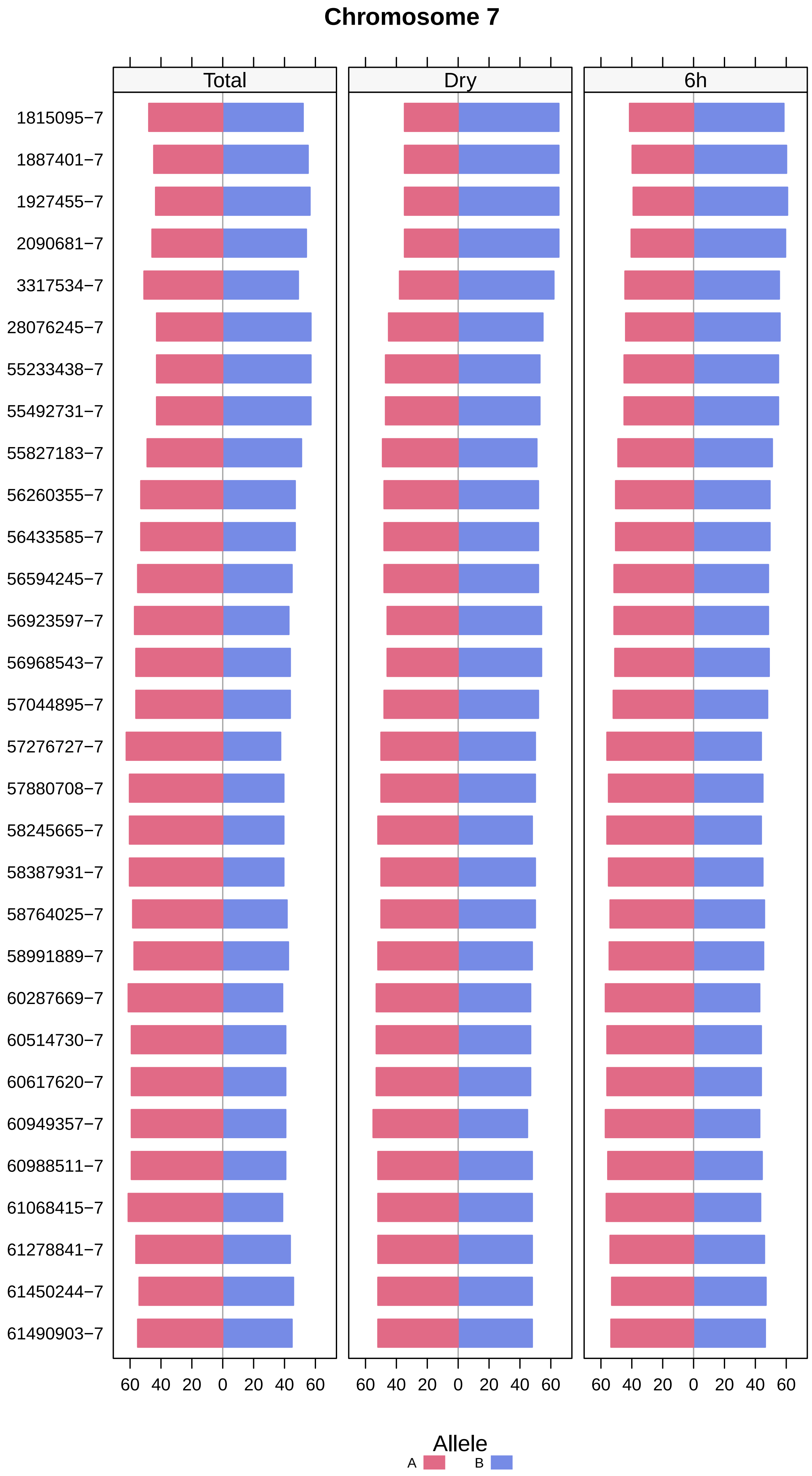
Chromosome 7
Total
Dry
6h
1815095−7
1887401−7
1927455−7
2090681−7
3317534−7
28076245−7
55233438−7
55492731−7
55827183−7
56260355−7
56433585−7
56594245−7
56923597−7
56968543−7
57044895−7
57276727−7
57880708−7
58245665−7
58387931−7
58764025−7
58991889−7
60287669−7
60514730−7
60617620−7
60949357−7
60988511−7
61068415−7
61278841−7
61450244−7
61490903−7
60 40 20 0 20 40 60
Allele
A
B

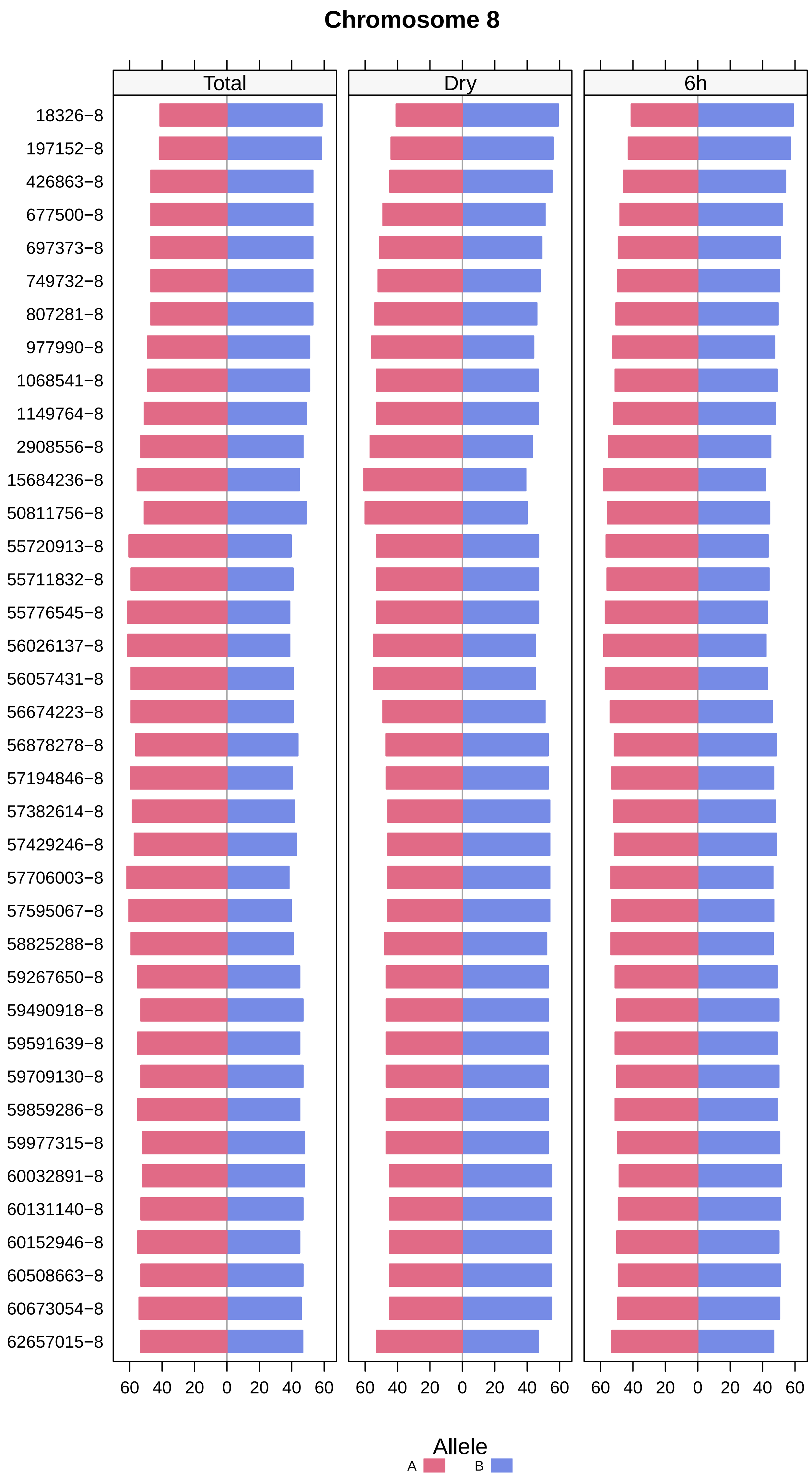
Chromosome 8
Total
Dry
6h
18326−8
197152−8
426863−8
677500−8
697373−8
749732−8
807281−8
977990−8
1068541−8
1149764−8
2908556−8
15684236−8
50811756−8
55720913−8
55711832−8
55776545−8
56026137−8
56057431−8
56674223−8
56878278−8
57194846−8
57382614−8
57429246−8
57706003−8
57595067−8
58825288−8
59267650−8
59490918−8
59591639−8
59709130−8
59859286−8
59977315−8
60032891−8
60131140−8
60152946−8
60508663−8
60673054−8
62657015−8
60 40 20 0 20 40 60
Allele
A
B

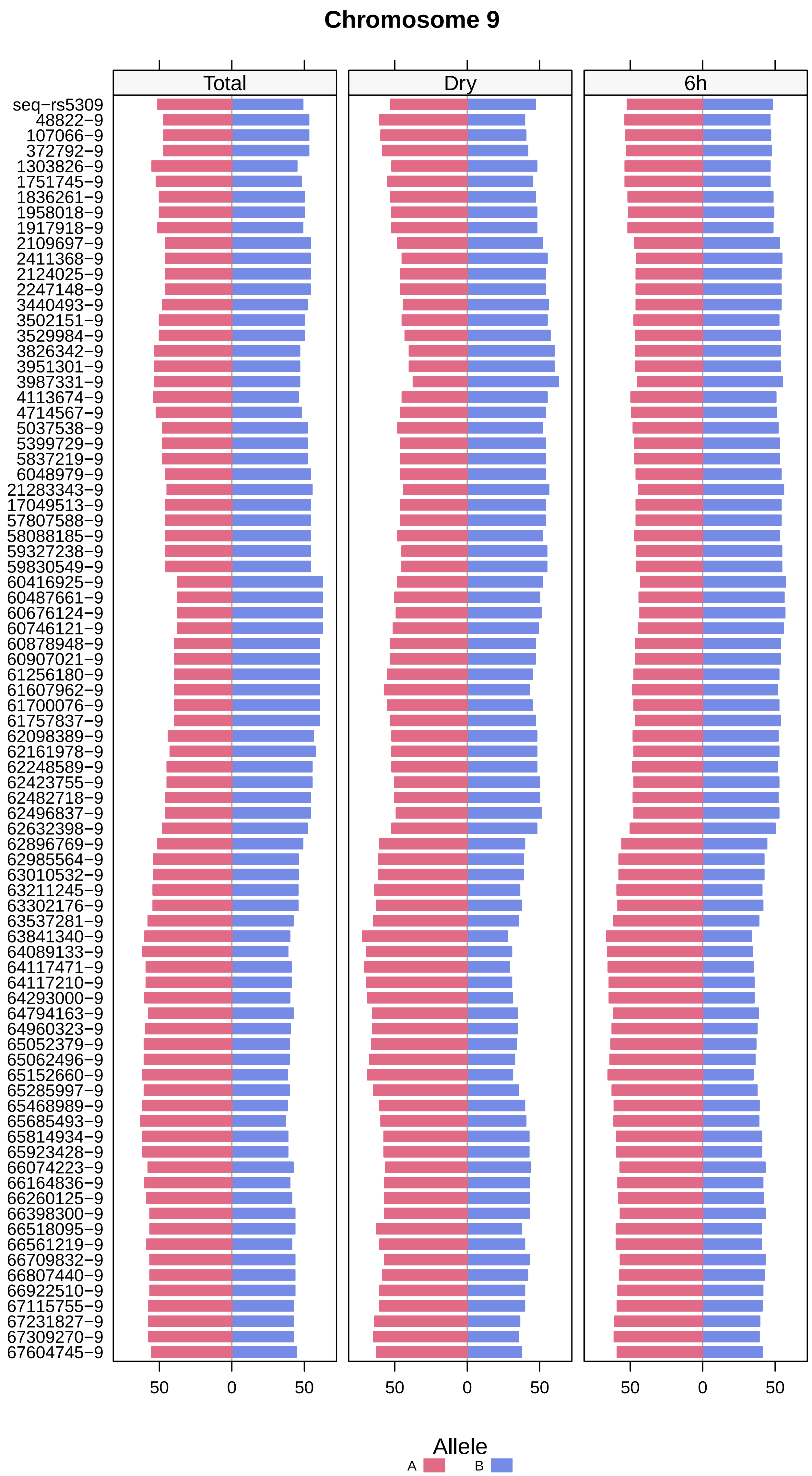
Chromosome 9
Total
Dry
6h
seq-rs5309
48822-9
107066-9
372792-9
1303826-9
1751745-9
1836261-9
1958018-9
1917918-9
2109697-9
2411368-9
2124025-9
2247148-9
3440493-9
3502151-9
3529984-9
3826342-9
3951301-9
3987331-9
4113674-9
4714567-9
5037538-9
5399729-9
5837219-9
6048979-9
21283343-9
17049513-9
57807588-9
58088185-9
59327238-9
59830549-9
60416925-9
60487661-9
60676124-9
60746121-9
60878948-9
60907021-9
61256180-9
61607962-9
61700076-9
61757837-9
62098389-9
62161978-9
62248589-9
62423755-9
62482718-9
62496837-9
62632398-9
62896769-9
62985564-9
63010532-9
63211245-9
63302176-9
63537281-9
63841340-9
64089133-9
64117471-9
64117210-9
64293000-9
64794163-9
64960323-9
65052379-9
65062496-9
65152660-9
65285997-9
65468989-9
65685493-9
65814934-9
65923428-9
66074223-9
66164836-9
66260125-9
66398300-9
66518095-9
66561219-9
66709832-9
66807440-9
66922510-9
67115755-9
67231827-9
67309270-9
67604745-9
50 0 50
Allele
A
B

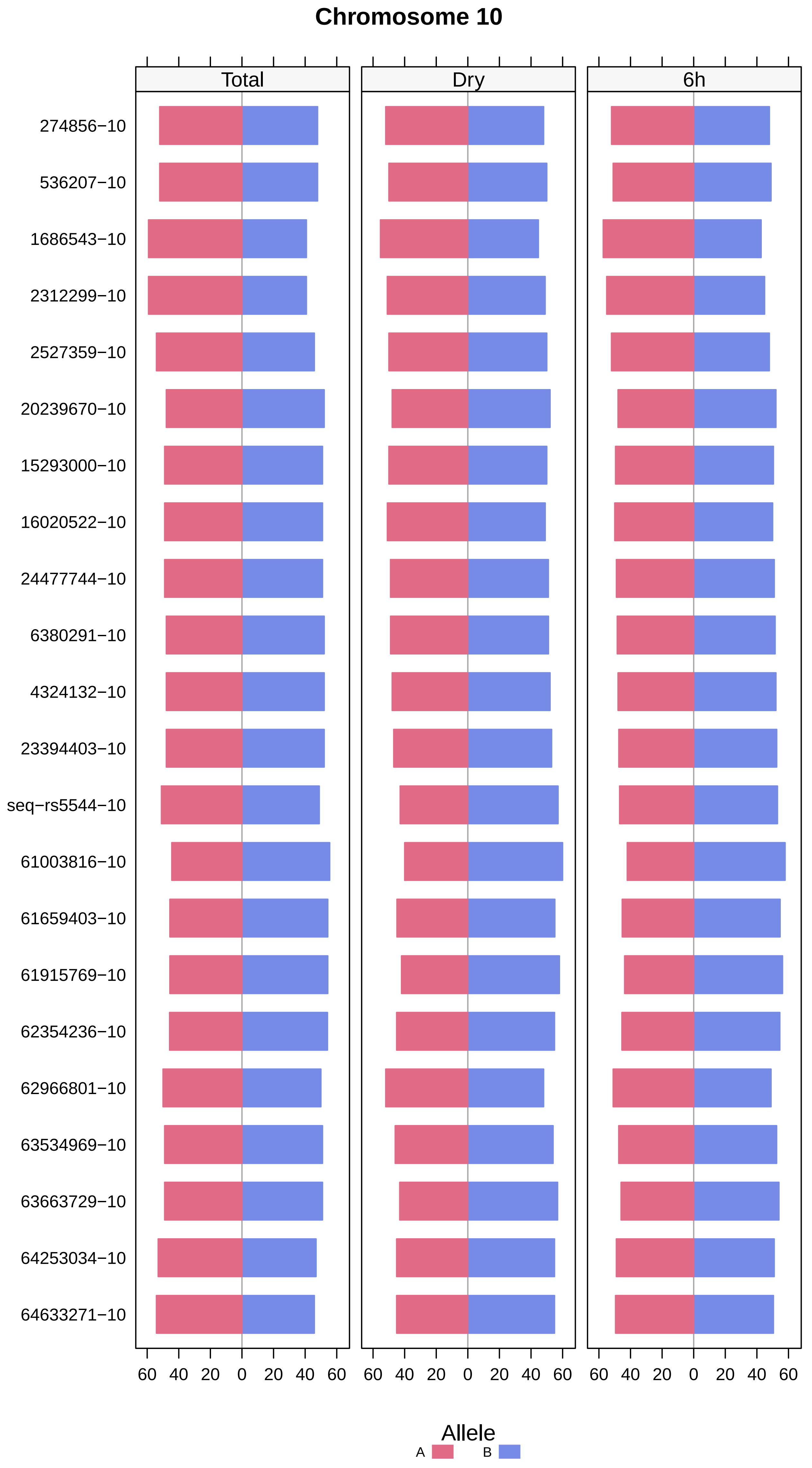
Chromosome 10
Total
Dry
6h
274856−10
536207−10
1686543−10
2312299−10
2527359−10
20239670−10
15293000−10
16020522−10
24477744−10
6380291−10
4324132−10
23394403−10
seq−rs5544−10
61003816−10
61659403−10
61915769−10
62354236−10
62966801−10
63534969−10
63663729−10
64253034−10
64633271−10
60 40 20 0 20 40 60
Allele
A
B

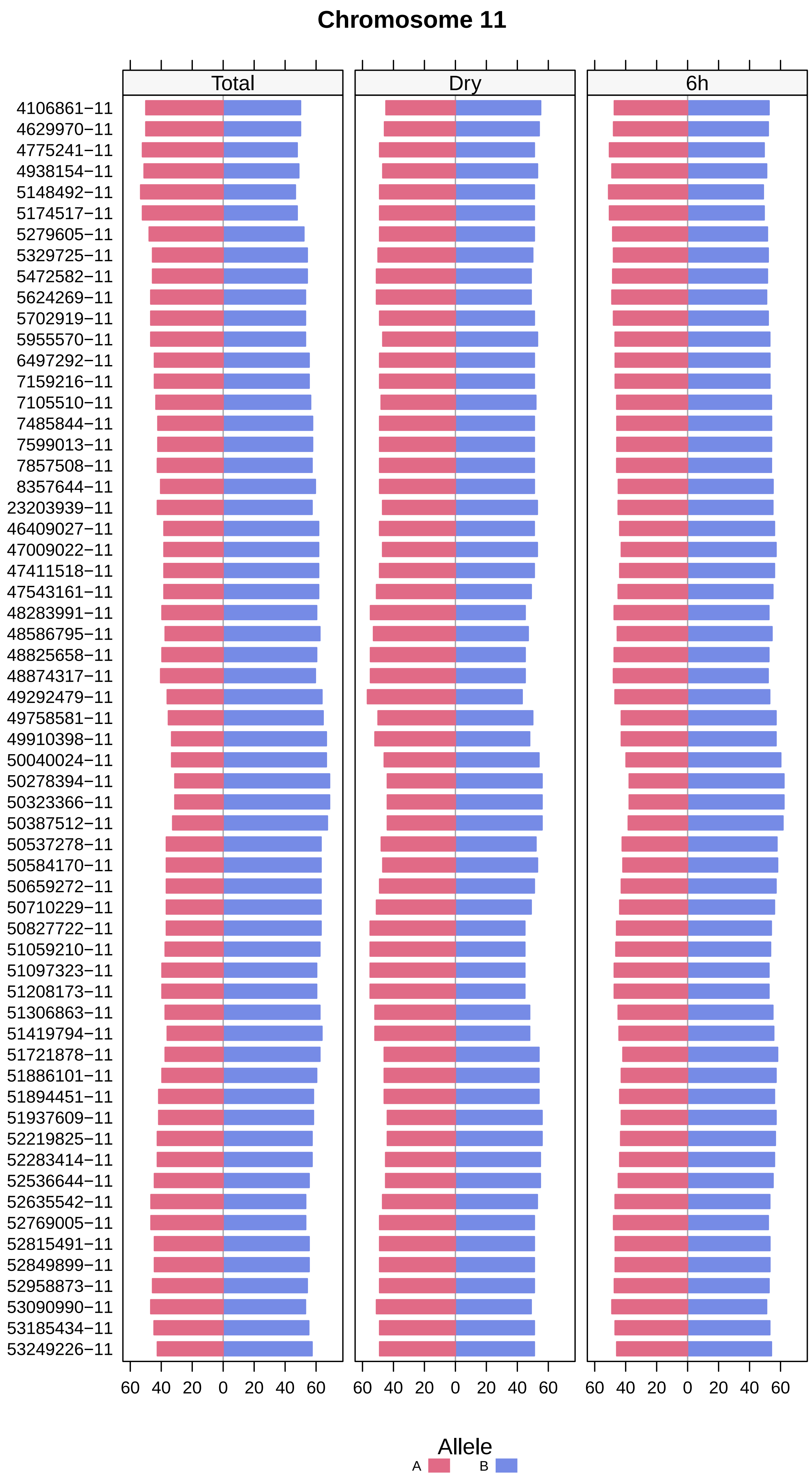

Chromosome 11
Total
Dry
6h
4106861−11
4629970−11
4775241−11
4938154−11
5148492−11
5174517−11
5279605−11
5329725−11
5472582−11
5624269−11
5702919−11
5955570−11
6497292−11
7159216−11
7105510−11
7485844−11
7599013−11
7857508−11
8357644−11
23203939−11
46409027−11
47009022−11
47411518−11
47543161−11
48283991−11
48586795−11
48825658−11
48874317−11
49292479−11
49758581−11
49910398−11
50040024−11
50278394−11
50323366−11
50387512−11
50537278−11
50584170−11
50659272−11
50710229−11
50827722−11
51059210−11
51097323−11
51208173−11
51306863−11
51419794−11
51721878−11
51886101−11
51894451−11
51937609−11
52219825−11
52283414−11
52536644−11
52635542−11
52769005−11
52815491−11
52849899−11
52958873−11
53090990−11
53185434−11
53249226−11
60 40 20 0 20 40 60
Allele
A
B

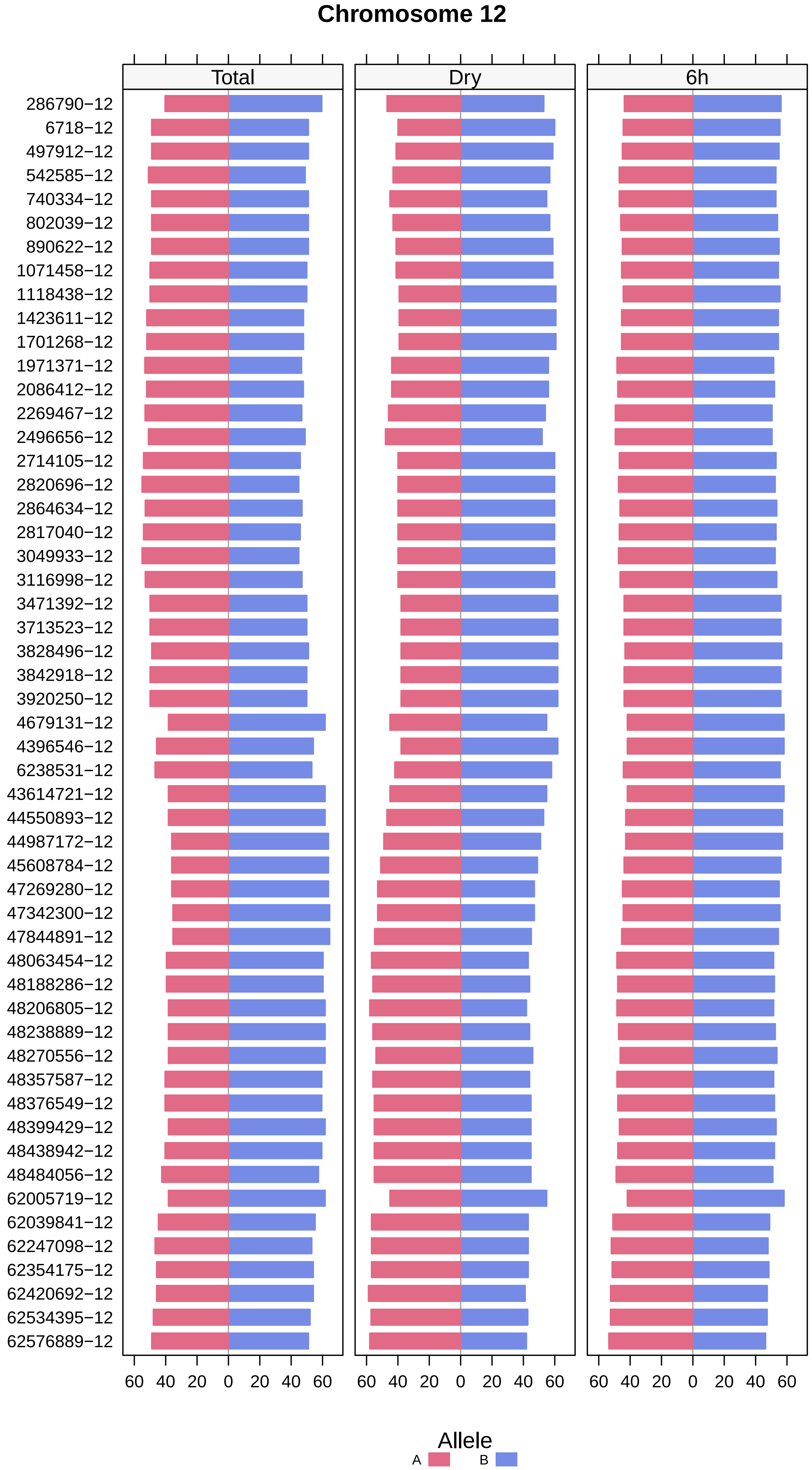
Chromosome 12
Total
Dry
6h
286790−12
6718−12
497912−12
542585−12
740334−12
802039−12
890622−12
1071458−12
1118438−12
1423611−12
1701268−12
1971371−12
2086412−12
2269467−12
2496656−12
2714105−12
2820696−12
2864634−12
2817040−12
3049933−12
3116998−12
3471392−12
3713523−12
3828496−12
3842918−12
3920250−12
4679131−12
4396546−12
6238531−12
43614721−12
44550893−12
44987172−12
45608784−12
47269280−12
47342300−12
47844891−12
48063454−12
48188286−12
48206805−12
48238889−12
48270556−12
48357587−12
48376549−12
48399429−12
48438942−12
48484056−12
62005719−12
62039841−12
62247098−12
62354175−12
62420692−12
62534395−12
62576889−12
60 40 20 0 20 40 60
Allele
A
B

Supplemental Figure 1. Intelligent allocation of 100 RILs population to two sub-populations. The core population of 100 RILs was divided in two subpopulations (i.e. 50 RILs for dry and 50 RILs for 6h imbibed seeds) optimized for the distribution of parental alleles using the R package DesignGG, aiming at the most accurate estimate of G and G:E effects.
