# Supplementary material for: Metabolomic analysis of tomato seed germination
Source: Metabolomics. 2017 Oct 23;13(12):145. doi: 10.1007/s11306-017-1284-x (PMC5653705; doi:10.1007/s11306-017-1284-x)
Supplement: Supplementary file 15 — Supplemental table 7. List of top 20 signature metabolites ranked according to the strength of the canonical correlation (G max Oxidative Stress; 300mM H 2 O 2 ) (DOCX 13 KB) [file 11306_2017_1284_MOESM15_ESM.docx]

**Supplemental table S7.** List of top 20 signature metabolites ranked according to the strength of the canonical correlation (G_max_ Oxidative Stress; 300mM H_2_O_2_ ).

| **Metabolites** | **COR** | **PV** |
| --- | --- | --- |
| RI_1379 | 0.55 | 4.33E-09 |
| Fructose | 0.54 | 4.51E-09 |
| RI_1957 | 0.54 | 4.84E-09 |
| RI_2866 | 0.53 | 1.14E-08 |
| Hexonic acid | 0.53 | 1.34E-08 |
| RI_2442 | 0.53 | 1.78E-08 |
| Hypotaurine | 0.47 | 5.76E-07 |
| Alpha-hydroxybutyrate | 0.46 | 1.16E-06 |
| Shikimate | 0.46 | 1.20E-06 |
| RI_2882 | 0.46 | 1.38E-06 |
| RI_2470 | 0.43 | 7.85E-06 |
| RI_1867 | 0.43 | 7.85E-06 |
| Glucuronate | 0.40 | 3.38E-05 |
| RI_2939 | 0.40 | 3.75E-05 |
| RI_1703 | 0.39 | 5.60E-05 |
| C16:0 | 0.38 | 9.92E-05 |
| Allantoin | 0.35 | 0.000282 |
| RI_2087 | 0.35 | 0.000353 |
| Xylofuranose | 0.34 | 0.000449 |
| Guanosine | 0.28 | 0.004237 |
